# MiR-130b modulates the invasive, migratory, and metastatic behavior of leiomyosarcoma

**DOI:** 10.1371/journal.pone.0278844

**Published:** 2023-01-26

**Authors:** Laura S. Danielson, Maria V. Guijarro, Silvia Menendez, Brett Higgins, Qiang Sun, Khushbakhat Mittal, Dorota A. Popiolek, Michael Overholtzer, Glyn D. Palmer, Eva Hernando

**Affiliations:** 1 Department of Pathology, NYU Grossman School of Medicine New York, NY, United States of America; 2 Department of Orthopaedics and Sports Medicine, University of Florida, Gainesville, FL, United States of America; 3 Cell Biology Program, Memorial Sloan-Kettering Cancer Center, New York, NY, United States of America; Hokkaido University: Hokkaido Daigaku, JAPAN

## Abstract

Leiomyosarcoma (LMS) is an aggressive, often poorly differentiated cancer of the smooth muscle (SM) lineage for which the molecular drivers of transformation and progression are poorly understood. In microRNA (miRNA) profiling studies, miR-130b was previously found to be upregulated in LMS vs. normal SM, and down-regulated during the differentiation of mesenchymal stem cells (MSCs) into SM, suggesting a role in LMS tumor progression. In the present study, the effects of miR-130b on human LMS tumorigenesis were investigated. Stable miR-130b overexpression enhanced invasion of LMS cells *in vitro*, and led to the formation of undifferentiated, pleomorphic tumors *in vivo*, with increased growth and metastatic potential compared to control LMS cells. *TSC1* was identified as a direct miR-130b target in luciferase-3’UTR assays, and shRNA-mediated knockdown of *TSC1* replicated miR-130b effects. Loss-of-function and gain-of-function studies showed that miR-130b levels regulate cell morphology and motility. Following miR-130b suppression, LMS cells adopted a rounded morphology, amoeboid mode of cell movement and enhanced invasive capacity that was Rho/ROCK dependent. Conversely, miR-130b-overexpressing LMS cells exhibited Rho-independent invasion, accompanied by down-regulation of Rho-pathway effectors. In mesenchymal stem cells, both miR-130b overexpression and *TSC1* silencing independently impaired SM differentiation *in vitro*. Together, the data reveal miR-130b as a pro-oncogenic miRNA in LMS and support a miR-130b-*TSC1* regulatory network that enhances tumor progression via inhibition of SM differentiation.

## Introduction

Leiomyosarcoma (LMS) is an aggressive malignancy of smooth muscle (SM) origin for which the molecular basis of transformation and mechanisms of progression are mostly unknown. Given the diversity of anatomical locations of SM tissues throughout the body, LMS can arise in various regions; however, the most common sites are the smooth muscle lining of the uterus (myometrium, MM) and the retroperitoneal cavity. Although tumors from different locations are histologically similar, they often have distinct clinical courses and response to chemotherapy. While studies have shown that LMS of cutaneous origin have a more favorable outcome [1], LMS of deep tissue origin, such as retroperitoneal tumors, are highly aggressive, commonly recur after resection, and despite therapeutic attempts, less than 50% of patients survive 5 years [2]. Therefore, further understanding of the mechanisms contributing to LMS pathobiology is needed to develop more efficacious therapies that improve patient outcome.

Molecular features of soft tissue LMS have been investigated using cytogenetic and genomic approaches to reveal multiple gene alterations and complex karyotypes [3]. Comparative genomic hybridization (CGH) studies have demonstrated broad chromosomal losses and gains [4], with altered regions encompassing > 2000 genes [5]. Integrative, large-scale genomic analysis have also been performed to distinguish LMS from other soft tissue sarcomas, and identify characteristic profiles of gene, DNA copy number, mutation data and methylation [6–10]. Further insights into tumorigenic mechanisms have been gained through gene profiling studies that identify LMS subtypes based on their metastatic potential [11], clinical outcome [8], and response to chemotherapy treatment [12]. While these advances have generated vital information to better guide LMS diagnosis and prognosis, the molecular mechanisms of leiomyosarcomagenesis remain largely unknown, necessitating a better understanding of the underlying disease pathology.

MicroRNAs are small, non-coding RNAs that exert important roles in a variety of biological processes by negative regulation of their mRNA targets [13]. MiRNAs are essential for the development of nearly all tissue and organ systems including SM [14, 15] and play a significant role in all aspects of tumorigenesis, from initiation to metastasis [16, 17]. Among sarcoma subtypes, miRNA profiling has been used to aid in tumor classification [18–21] and identify potential diagnostic and prognostic biomarkers [22–24]. While several miRNAs have been identified with tumor suppressive or oncogenic functions in a range of sarcomas [25, 26], miRNA-mediated regulation of tumorigenic pathways in LMS has yet to be described.

In an effort to better understand LMS pathogenesis, we previously performed profiling studies to identify candidate miRNAs involved in cellular transformation and SM differentiation [14]. Among these, miR-130b was found to be both overexpressed in LMS patient samples vs normal myometria and downregulated during mesenchymal stem cell (MSC) differentiation into the SM lineage. Based on these findings we hypothesize a functional role for miR-130b in LMS and SM differentiation. Our data support a pro-oncogenic role for miR-130b in LMS, via direct inhibition of the tumor suppressor *TSC1* and blockade of SM differentiation.

## Materials and methods

### Clinical samples

**Fig 1:**
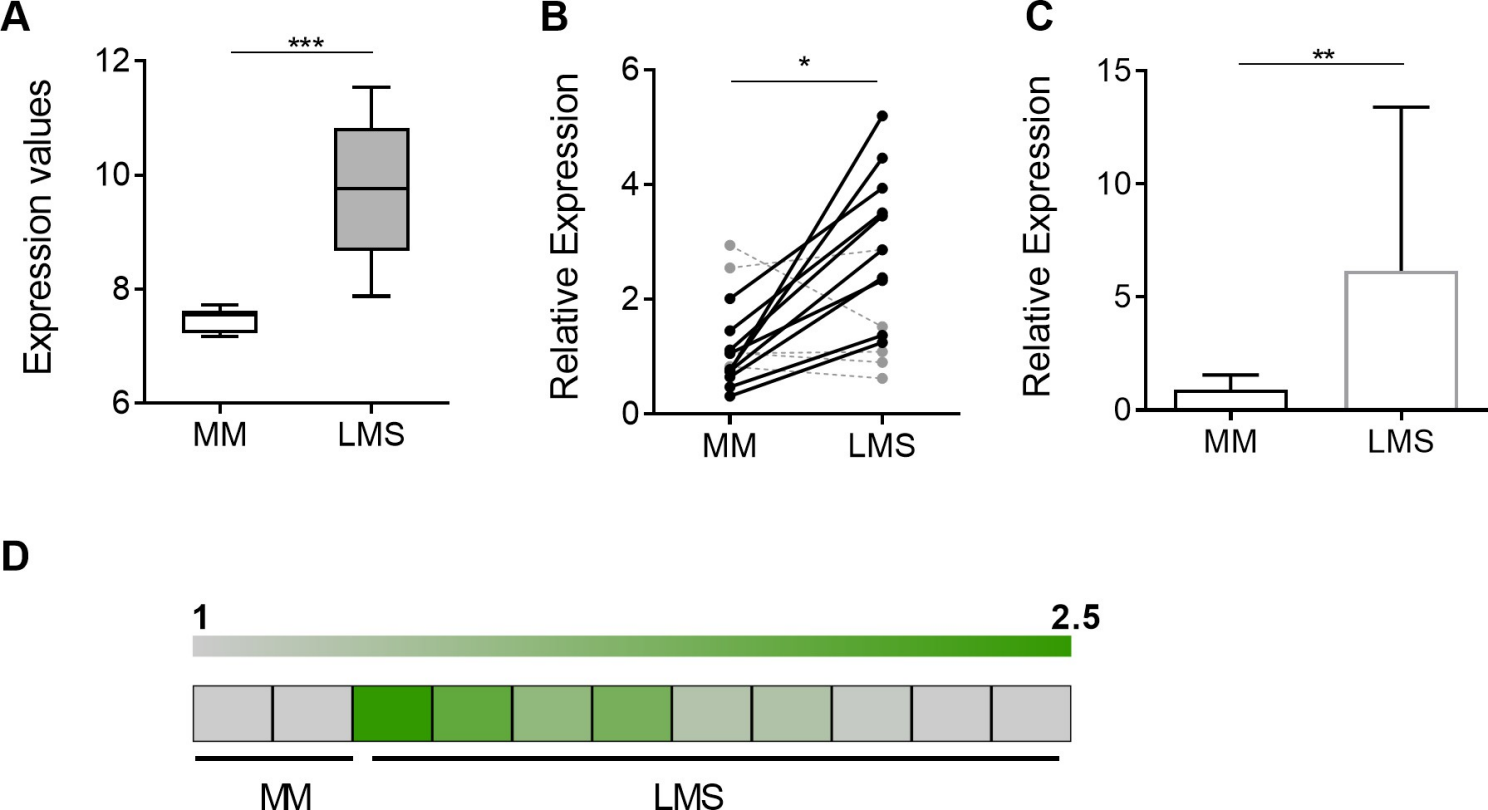
MiRNA-130b expression is upregulated in LMS. (A) Relative miR-130b levels, determined by miRNA array profiling of myometrial (MM; n = 10) and leiomyosarcoma (LMS; n = 10) tissues adapted from previously published data [14]. (B) Relative miR-130b levels, measured by RT-qPCR, in separate cohort of matched MM and LMS tissues (n = 15). Expression values are normalized to the average miR-130b expression levels for the whole MM group. (C) Relative miR-130b levels, measured by RT-qPCR, in a third cohort of non-matched MM (n = 19) and LMS (n = 13) tissues. (D) Gene amplification, determined by qPCR, of the *MiR-130b* locus in MM (n = 2) and LMS (n = 9) tissue samples, represented as a heat map. Average levels in normal MM (= 1) are indicated by grey color; amplified loci are represented by shades of green. Statistical analyses were performed by two-tailed students t-test; *P < 0.05, ** *P* < 0.005, *** *P* < 0.001.

Human tissue specimens for microarray analysis (Fig 1A) were collected at the time of surgery and snap frozen in liquid nitrogen and transferred to -80°C for storage. Approval to collect specimens was granted by MSKCC IRB protocol number #97–134. Methods and samples have been previously published [27]. For validation studies and assessment of genomic DNA amplification (Fig 1B–1D), archival formalin-fixed and paraffin-embedded (FFPE) specimens of normal myometrial (MM) and LMS tissues were obtained from repositories at Memorial Sloan Kettering Cancer Center (MSKCC), Bellevue and Northwestern University after Institutional Review Board approval. Matched MM and LMS were obtained under NYU IRB protocol H10457-01A. All human LMS tumor samples were of uterine origin and diagnosis of soft tissue uterine LMS was performed on core biopsy tissues by gynecologic specialty pathologists. MM tissues in non-patient matched samples were collected form patients undergoing surgical procedures for benign indications.

### Cell culture and SM differentiation

Human BM-MSCs were obtained from the Texas University Institute of Regenerative Medicine MSC Distribution Program (http://medicine.tamhsc.edu/irm/msc-distribution.html) and cultured as previously described [14]. SK-LMS1, SK-UT1 and 293T cell lines were purchased from ATCC (Manassas, VA, USA) and cultured in IMDM (LMS cells) or DMEM (293T) (Invitrogen, Carlsbad, CA, USA), supplemented with 10% fetal bovine serum (Cell Gro, Manassas, VA) and 100U/ml penicillin/streptomycin. Smooth muscle differentiation of BM-MSCs using TxA2 for myogenic induction was performed as previously described [14, 28]. Induction was performed by media supplementation with 1.0 μM of the TXA2 analog U46619 (Enzo Life Sciences, Farmingdale, NY, USA).

### Lentiviral transduction and transient transfection of miRNAs

Lentiviral expression plasmids for miRNA expression/knockdown or controls were obtained from System Biosciences (Palo Alto, CA, USA) as follows: MIRH empty (#CD511B-1), MIRH-SCR (#PMIRH000PA-1), MIRH-miR130b (#PMIRH130bPA-1), ZIP-SCR (#MZIP000-PA-1), ZIP-miR130b (#MZIP130b-PA-1). For shRNA-mediated knockdown studies, expression plasmids were obtained from Dharmacon (Lafayette, CO, USA) encoding scrambled, GIPZ-SCR (#RHS4346), or shTSC1 sequences (#RHS4430-99147968). Expression plasmids were co-transfected into 293T cells with the second-generation packaging plasmids pSPAX-2 (Addgene ID# 12260) and pMD2.G (Addgene ID# 12259) using Lipofectamine 2000 transfection reagent (Invitrogen), and viral supernatants were harvested 48 h later and used directly for cell transduction. For infection, cell monolayers were transduced with viral supernatants 1-3x in the presence of polybrene (8 μg/ml).

To investigate miR-130b on SM myogenesis, hMSCs were transiently transfected with control (Dharmacon; #CN-001000-01) or miR-130b-3p (#C-300660-05) oligonucleotide miRNA mimics (50 nM) using Lipofectamine, 24 h prior to induction of differentiation. For *TSC1* inhibition, non-targeting control siRNA (Dharmacon; D-0017810-10) or si*TSC1* (#L-003028-00-0005) were transfected (100 nM) under the same conditions. For gene expression profiling studies, SK-LMS1 and SK-UT1 were transfected with control or miR-130b-3p mimics (50 nM) and cells harvested for RNA preparation 36 h later.

### RNA and gene expression

Total RNA was extracted from cell cultures using the miRNeasy Mini Kit (Qiagen, Germantown, MD, USA). For determination of miRNA levels, qRT-PCR was performed on 500 ng total RNA using Taqman reverse transcription reagents and miRNA assay (Applied Biosystems, Foster City, CA, USA) using the small RNA, RNU44 for normalization. For assay of mRNA levels, qRT-PCR was performed using the Superscript III reverse transcription kit (Invitrogen) and the FastStart SYBR Green MasterMix (Roche, Branchburg, NJ, USA) with gene-specific PCR primers designed from human sequences (S1 Table).

For quantification of miRNA and genomic amplification in clinical specimens, miRNA or genomic DNA were isolated from formalin-fixed, paraffin-embedded MM and LMS tissues using miRNeasy FFPE RNA and DNA FFPE extraction kits, respectively (Qiagen). Genomic amplification was analyzed by qPCR as above, and values were normalized to *B2M* genomic DNA levels using gene-specific primers (S1 Table).

### Gene array profiling

Gene expression profiling of two LMS cell lines (SK-LMS1 and SK-UT1) transfected with control or miR-130b oligonucleotide mimics was obtained with the Affymetrix Genechip system (ThermoFisher, Waltham, MA, USA). Cells were harvested 36 h post transfection and 100 ng of total RNA were used to prepare cRNA following the Affymetrix 3’IVT Express Kit labeling protocol. Standardized array processing procedures including hybridization, fluidics processing and scanning of the Affymetrix HG-U133 Plus 2.0 arrays were performed according to the manufacturer’s instructions. GeneSpring GX11 software (Agilent Technologies, Santa Clara, CA, USA) was used to normalize the raw data (Affymetrix CEL files) using the Robust Multichip Average algorithm (RMA) [29], to filter and perform differential expression analyses using T-test statistics (*P* < 0.05, alpha level) and fold-change thresholding (>33% reproducible change) using the volcano plot feature. Lists of modulated genes were overlapped using the Venn diagram feature to identify transcripts altered in both cell lines, and further filtered by *in silico* miRNA target prediction (www.targetscan.com).

### Western blot analyses

Cells were harvested for protein extraction and immunoblotting as described previously [14]. Primary antibodies used were for detection of β-Tubulin (#T9026, Sigma, St Louis, MO, USA), TSC1/hamartin (#370400, Life Technologies, Carlsbad, CA, USA), phospho-myosin light chain, (#3671, Cell Signaling, Danvers, MA, USA), and Rho-GTP (#BK036, Cytoskeleton, Inc Denver, CO, USA).

### Immunofluorescence

For phalloidin staining, as-miR-130b or control transduced cells were seeded on cover slips overnight, fixed with 4% formaldehyde, permeabilized with 0.1% triton, and incubated with Phalloidin (#A34055, Invitrogen), followed by DAPI counterstaining. Cultures were then mounted with ProLong Gold mounting reagent (Invitrogen), imaged using a Leica SP5 confocal microscope (Leica, Wetzlar, Germany) and analyzed using image J software (NIH, Bethesda, MD, USA)

### Cell migration assays

Wound healing ‘scratch’ assays were performed by seeding cells at 80% confluence and scratching the surface of the well through the center using a 1 ml pipette tip after 24 h. Images of the ‘wound gap’ were taken under phase-contrast and fluorescence microscopy over a 24 h time period and measured using Image J software, to determine the extent of closure. For time-lapse videography, 5 x 10^3^ cells plated into 6-well glass bottom dishes and videomicroscopy images were taken every 7 minutes for 2 h. Cell positions (n = 25, for each cell type) were tracked manually and marked by the centroid of the nucleus in each frame. Cell migration speed was determined using ImageJ software. All analyses were performed in a blinded fashion.

### Cell invasion assays

Transwell invasion assays were performed using 8 μm pore inserts coated with 100 μg/ml fibronectin (Sigma) for 1 h, followed by blocking of insert membrane with 2.5% BSA for 30 minutes. For matrigel coated inserts, a 1:40 dilution of matrigel (Corning, Corning, NY, USA) was incubated on the membrane for 30 minutes and then washed with serum-free media before seeding cells. Inserts were seeded at 2 x 10^4^ cells in IMDM (serum-free) and allowed to invade for 16–20 h. Following removal of non-invasive cells, the bottom of the insert membrane was fixed with 0.1% glutaraldehyde, stained with crystal violet and analyzed by phase contrast microscopy to determine the number of cells per field. For ROCK inhibition, LMS1 cells were pre-incubated for 3 h with either vehicle or Y27632 (#04–0012; StemGent, Cambridge, MA, USA) prior to seeding and maintained in the presence of the inhibitor for the experiment duration.

### *In vivo* xenotransplantation

All animal experiments were performed in accordance with the NYU IACUC approved protocols (#061108–03 and #100108–01). To determine the tumor growth and metastatic properties of LMS cell lines, 5 x 10^6^ cells were combined 1:1 with matrigel and injected subcutaneously into the flanks of 6-weeks old immunocompromised mice (NOD.Cg-Prkdcscid Il2rgtm1Wjl/SzJ, #05557 Jackson Labs, Bar Harbor, ME, USA). Once tumors were palpable, tumor length and width were measured every other day and volume was calculated using the following formula: a^2^*b/2, where *a* is the width and *b* is the length. After 7 weeks, primary tumors were excised, weighed and processed for paraffin histology according to standard protocols. Cell proliferation was assessed by immunostaining for Ki67 (Neomarkers, Portsmouth, NH, USA) and counterstaining with hematoxylin. Lung metastasis was assessed by fluorescence microscopy of whole lung tissue recovered at endpoint, for quantification of GFP+ foci. Primary tumors were graded according to WHO classification for soft tissues [30].

### Luciferase reporter assay

Human-3’UTR luciferase reporter constructs (except *TSC1*) were purchased from Switchgear Genomics (Menlo Park, CA, USA). For generation of a *TSC1*-3’UTR reporter construct, genomic DNA containing a portion of the *TSC1*-3’UTR sequence was PCR-amplified using the primers *TSC1*-3’UTR-Fw (XhoI): 5’ CCCTCGAGGGGTTTGGGCAGAGCGAAGACC−3’; and *TSC1*-3’UTR-Rv (NotI): 5’-TTGCGGCCGCAAGCCACTCATTGAGGAAGAGC-3’, subcloned into the pCR-II vector using TOPO-TA cloning kit (ThermoFisher) for sequencing, and then cloned into a pLS-Empty Vector (Switchgear genomics) by XhoI/NotI ligation. For generation of luciferase constructs containing individual miR-130b target sites and target site mutants, double stranded DNA fragments (~80 bp), corresponding to regions of *TSC1* 3’UTR genomic DNA, were synthesized using Genscript Gene Synthesis Services (Genscript, Piscataway NJ) and cloned downstream of luciferase into the pLS-Empty Vector by Nhe1/XhoI ligation. The partial 3’UTR sequences are shown in S2 Table. For functional target validation, the 3’UTR luciferase constructs were cotransfected with miRNA mimics (miR-130b or SCR; 50 nM) or hairpin inhibitors (miR-130b HI or cel67 HI; 50 nM) as per the manufacturers’ protocol. Luciferase activities were determined 24 h post-transfection at 595 nm and values were normalized to mock (no miRNA) controls. Experiments were performed a minimum of 3 times, with 4 technical replicates per experiment.

### Statistical analysis

Statistical significance was determined using GraphPad Prism Software. Unpaired t-tests were used to compare single data points. Statistical significance in all analyses was defined as *P* < 0.05. *In vitro* tumorigenic assays were performed a minimum of three times with at least two different stably transduced cell lines. For SM differentiation assays, data are shown for a single experiment representative of at least 3 experiments performed. Unless otherwise indicated, data presented is a representative experiment.

## Results

### MiRNA-130b is overexpressed in LMS

We previously performed miRNA profiling studies to identify miRNAs associated with LMS pathogenesis. MiR-130b was identified as one of eight miRNAs found to be down-regulated in SM differentiation and upregulated in LMS tissue samples, suggesting its possible role as a pro-oncogenic miRNA in LMS tumor progression [14]. MiR-130b expression levels, determined from miRNA microarrays, were found to be increased 1.3-fold in LMS patient tissues (n = 10) relative to normal, myometrial (MM) tissue controls (n = 10) (*P < 0*.*001*; Fig 1A). To validate these findings, miR-130b levels were further investigated in a separate, patient-matched cohort of MM and LMS tissues by RT-qPCR (n = 15) (Fig 1B). MiR-130b levels were increased [14] in 10 out of 15 pairs (fold increase: 2.56 ± 0.36; *P* = 0.003) (Fig 1B). Validation was also performed by RT-qPCR in a third independent cohort of (non-matched) patient samples of normal MM (n = 19) and LMS (n = 13) tissues and revealed significant elevation of miR-130b levels (fold change: 6.8 ± 8, *P* = 0.003) in tumor samples (Fig 1C). Amplification of the *MiR-130b* locus was then performed by qPCR following genomic DNA extraction of formalin fixed paraffin embedded (FFPE) MM and LMS clinical specimens (Fig 1D). Gene amplification of the *MiR-130b* locus was detected in 4 out of 9 LMS samples (*P* < 0.05, Fig 1D). Together, these data support a significant upregulation of miR-130b in LMS, which may arise from genomic amplification of the *MiR-130b* locus in a subset of patients.

### MiR-130b promotes LMS cell invasion *in vitro* and metastasis *in vivo*

**Fig 2:**
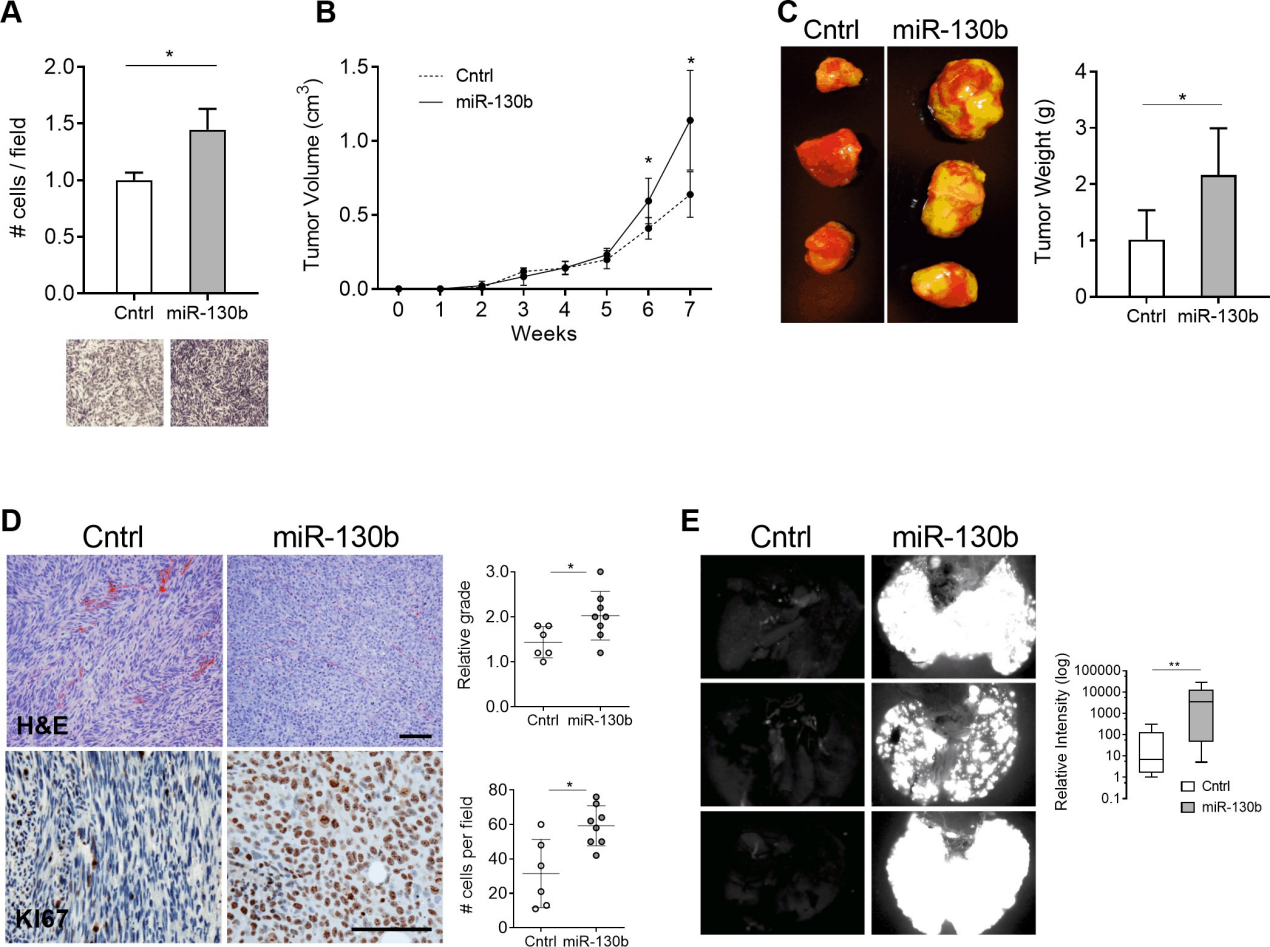
MiR-130b overexpression enhances LMS invasion and metastasis. (A) Transwell assay showing relative invasion of miR-130b overexpressing LMS1 cells and control 18 h after seeding (n = 3, per group). Images show crystal violet staining of membrane inserts. Cell counts were normalized to the control group. (B) Primary tumor growth following subcutaneous injection of control (n = 6) and miR-130b (n = 8) transduced LMS1 cells in immunocompromised mice. (C) Representative images of primary tumors at the 7-weeks endpoint (*left panel*). Mean weight of resected primary tumors at endpoint for control (n = 6) and miR-130b (n = 8) groups (*right panel*). (D) H&E (*top panels*) and Ki67 (*bottom panels*) stained primary tumors removed at endpoint. Scale bar: 100 μm. Plots show histological grading of tumor architecture (1 = fascicular; 3 = storiform) (*top*), and number of Ki67 positive cells per field (*bottom*). (E) Fluorescent images of GFP positive nodules in whole lungs recovered at endpoint from control and miR-130b transduced LMS1 cells. Box plot (*right*) depicts quantification of GFP signal intensity in lungs of control (n = 6) and miR-130b (n = 8) groups. Statistical analyses were performed by two-tailed students t-test; *P < 0.05, ** *P* < 0.005.

To determine if miR-130b overexpression affects the tumorigenic properties of LMS, *in vitro* oncogenic assays were performed using the LMS cell line, SK-LMS-1 (hereafter LMS1) stably transduced with a dual expression lentiviral vector encoding miR-130b and GFP (S1 Methods, S1A Fig). MiR-130b overexpression had no significant effects on cell proliferation in 2D cultures, colony formation, migration, growth in suspension, or ability to form sarcospheres, compared to LMS1 controls expressing a scrambled, non-targeting miRNA (SCR) (S1B–S1F Fig). However, in transwell invasion assays, miR-130b overexpression led to a significant increase in LMS1 invasive capacity compared to miRNA controls (*P* = 0.016) (Fig 2A).

To investigate whether miR-130b enhances LMS tumorigenesis *in vivo*, cells were xenotransplanted in immunocompromised mice. At endpoint (7 weeks), miR-130b overexpressing tumors exhibited significant increases in volume (*P* = 0.006) (Fig 2B) and weight (*P* = 0.012) (Fig 2C) relative to miRNA controls. H&E staining of resected tumors demonstrated a striking change in cellular architecture following miR-130b overexpression (Fig 2D, *top panels*). While controls displayed a fascicular pattern of cells with cigar shaped nuclei, common histological features of LMS, miR-130b overexpressing tumors presented a storiform appearance with pleomorphic nuclei, characteristic of pleomorphic undifferentiated sarcomas. Grading of tumors revealed a higher score in the miR-130b group (2.0 ± 0.5) compared to controls (1.4 ± 0.3) (*P* = 0.037) (Fig 2D). Immunohistochemical detection of the proliferation antigen, Ki67, also revealed a 1.9-fold increase in miR-130b overexpressing tumors (*P* = 0.0036) (Fig 2D, *bottom panels*) consistent with increased tumor growth.

Metastatic burden at endpoint was also assessed in resected lungs of xenotransplanted mice by monitoring GFP expression. *Ex vivo* fluorescent imaging revealed a marked increase in GFP positive lung foci in mice with miR-130b overexpressing tumors compared to controls (Fig 2E). Fluorescence intensity levels were >100 fold higher in the miR-130b group versus controls indicating a greatly enhanced metastatic capacity (*P* = 0.029) (Fig 2E, *right panel*). Altogether, the data demonstrates that miR-130b promotes an aggressive pleomorphic tumor phenotype in LMS1 cells, by accelerating tumor growth and enhancing metastatic potential.

### MiRNA-130b directly targets the tumor suppressor *TSC1*, and regulates multiple effectors of Rho signaling

**Fig 3:**
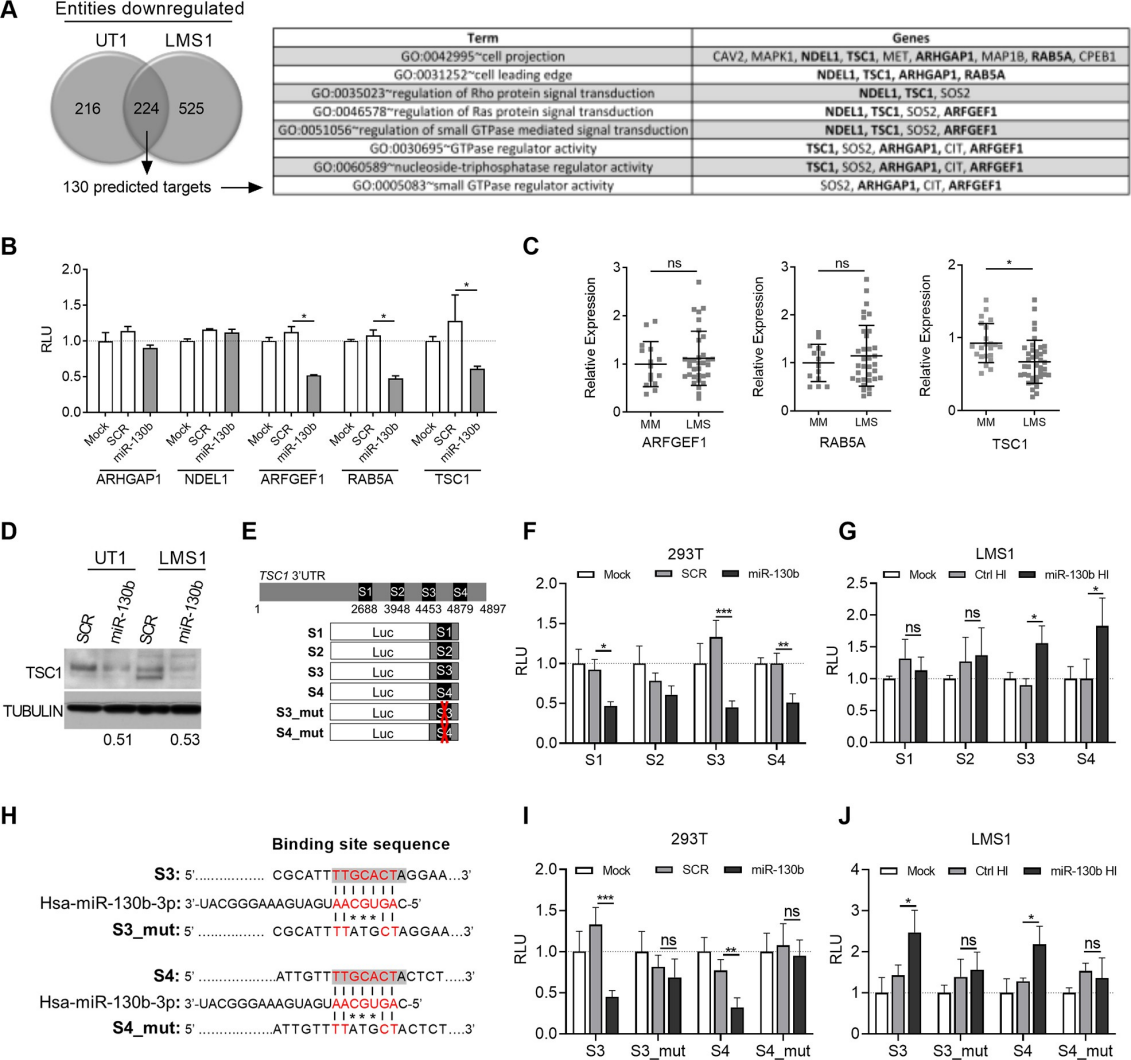
*TSC1* is a direct target of miR-130b and downregulated in LMS. (A) Number of transcripts showing significant downregulation (fold change: 1.33; *P* < 0.05) following gene array profiling of SK-LMS1 and SK-UT1 cell lines transfected with miR-130b or mimic controls (*left*). Profiling was performed in triplicate for each cell line. Gene ontology analysis was performed on shared downregulated genes identified as putative direct targets by target site prediction (130 total), revealing enrichment of pathways associated with cell motility and Rho GTPase signaling (*right*). (B) Luciferase-3’UTR reporter assay for validation of 5 candidate miR-130b targets selected from (A). Values represent relative luciferase units (RLU) in 293T cells following mock (no mimic), SCR or miR-130b mimic transfection (n = 4). Values are normalized to the mock control for each luciferase-3’UTR construct. Statistical analyses were performed by two-tailed students t-test; *P < 0.05 vs SCR controls. (C) Relative mRNA expression of validated targets, from (B), using a previously published dataset [27]. Note that only *TSC1* shows significant downregulation compared to normal MM. Statistical analyses were performed by two-tailed students t-test; *P < 0.05 vs MM. (D) TSC1 Western blot in UT1 and LMS1 cell lines 48 h after miR-130b or SCR control transfection. Numbers indicate relative TSC1 band intensities of miR-130b-transfected groups versus SCR controls after normalizing using tubulin levels. (E) S1-S4 luciferase reporter constructs for identification of functional miR-130b sites in the *TSC1* 3’UTR. (F) Relative luciferase activities of S1-S4 constructs in 293T cells following mock, SCR or miR-130b transfection (*as in panel B*) (n = 4). (G) Relative luciferase activities of S1-S4 constructs in LMS1 cells following mock, ctrl HI or miR-130b HI transfection (n = 4). (H) MiR-130b binding site sequences in wild type (S3 and S4) and mutant (S3_mut and S4_mut) constructs. Shaded text indicates predicted MiR-130b binding sites within the wild type sequences. Red text indicates seed region. (I) Relative luciferase activities of wild type and mutant S3 and S4 constructs in 293T cells following mock, SCR or miR-130b transfection (n = 4). (J) Relative luciferase activities of wild type and mutant S3 and S4 constructs in LMS1 cells following mock, ctrl HI or miR-130b HI transfection (n = 4). Statistical analyses (*F*, *G*, *I*, *J*) were performed by two-tailed students t-test; *P < 0.05, **P < 0.005, ***P < 0.001 vs SCR or Ctrl HI. Values are normalized to the mock controls for each luciferase construct.

To identify the tumorigenic pathways and mRNA targets regulated by miR-130b, gene expression profiling was performed on two LMS cell lines, LMS1 and SK-UT1 (hereafter, UT1) following transient transfection with miR-130b or miRNA mimic controls (SCR). Expression profiling of RNA extracted from LMS cells 36 h post-miRNA transfection identified 224 transcripts significantly down-regulated in both LMS1 and UT1 (fold change: 1.33, *P* < 0.05) (Fig 3A). Among the downregulated genes, 130 (58%) were predicted to be direct targets of miR-130b according to a publicly available algorithm (www.targetscan.org). Functional annotation of those potential targets revealed a set of genes involved in GTPase signaling, cell projection and leading edge, suggesting a role for miR-130b in the regulation of cell motility (Fig 3A). This was consistent with the observed modulatory effects of miR-130b overexpression on cell invasion and metastatic capacity *in vivo*.

To validate miR-130b-target gene interaction, luciferase constructs containing 3’-UTRs of 5 candidate genes selected as both, downregulated by miR-130b and predicted targets (Fig 3A), were cotransfected with miR-130b or mimic controls in HEK293 cells. *ARFGEF1*, *RAB5A*, and *TSC1* were found to be targets, as evidenced by significant repression of luciferase activity following transfection with miR-130b (*P* < 0.05) (Fig 3B). Among these, only *TSC1* was found to be significantly repressed in LMS tissues compared to normal MM, using a previously compiled dataset of LMS patient samples [27] (Fig 3C). *TSC1* was further supported as a target of miR-130b in LMS by repression of TSC1 levels (Fig 3D) following transient ectopic expression of miR-130b in UT1 and LMS1 compared to cells transduced with mimic controls. These data indicate that regulation of *TSC1* by miR-130b may contribute to the pathogenesis of LMS.

To further investigate the interaction of miR-130b and *TSC1*, target site prediction of the 3’UTR was performed using TargetScan and miRDB databases, which identified putative non-conserved sites at seed locations 2688 (S1) and 3948 (S2), and conserved sites at 4453 (S3) and 4879 (S4) (Fig 3E). Truncated portions of the 3’UTR, each containing a target site, were cloned into luciferase reporter constructs (Fig 3E). To identify which sites mediate the repressive activity of miR-130b, the constructs, S1-S4, were co-transfected with miR-130b mimic or SCR controls in 293T cells (Fig 3F). Three sites, S1, S3, and S4 were found to significantly downregulate luciferase activity in response to miR-130b (Fig 3F). To determine which of these sites mediate repressive activity in response to endogenous levels of miR-130b, each construct was cotransfected with a miR-130b- or ctrl-hairpin inhibitor (HI) in LMS1 cells (Fig 3G). MiR-130b HI cotransfection significantly increased luciferase activities of the S3 and S4 constructs relative to HI controls, indicating derepression of the miR-130b target sites following miR-130b inhibition (Fig 3G). To determine whether the S3 and S4 target sites mediate direct binding to miR-130b, two additional luciferase reporter constructs, S3_mut and S4_mut, were generated, each harboring a 3-bp mis-match mutation at positions +3 to +5 of the seed region (Fig 3H). In 293T cells, luciferase activities of S3_mut and S4_mut were not down-regulated in response to miR-130b mimic transfection, relative to SCR controls, in contrast to the wild type S3 and S4 constructs (Fig 3I), indicating a loss of sensitivity to miR-130b-mediated repression. Moreover, in LMS1 cells, luciferase activities of S3_mut and S4_mut were not upregulated by miR-130b HI transfection, contrary to the wild type constructs, indicating that sensitivity to endogenous regulation by miR-130b was lost (Fig 3J). In UT-1 cells, only the wild type S3 construct demonstrated significant upregulation of luciferase activity following miR-130b HI transfection (S3 Fig), suggesting variable sensitivities of S3 and S4 to binding of miR-130b among UT-1 and LMS cell types. Together, these findings indicate that the *TSC1* 3’UTR harbors two sites, S3 and S4, that can mediate the repressive activity of miR-130b in LMS cell lines, through direct binding of the microRNA.

### Independent silencing of *TSC1* replicates the pro-invasive and metastatic effects of miR-130b overexpression

**Fig 4:**
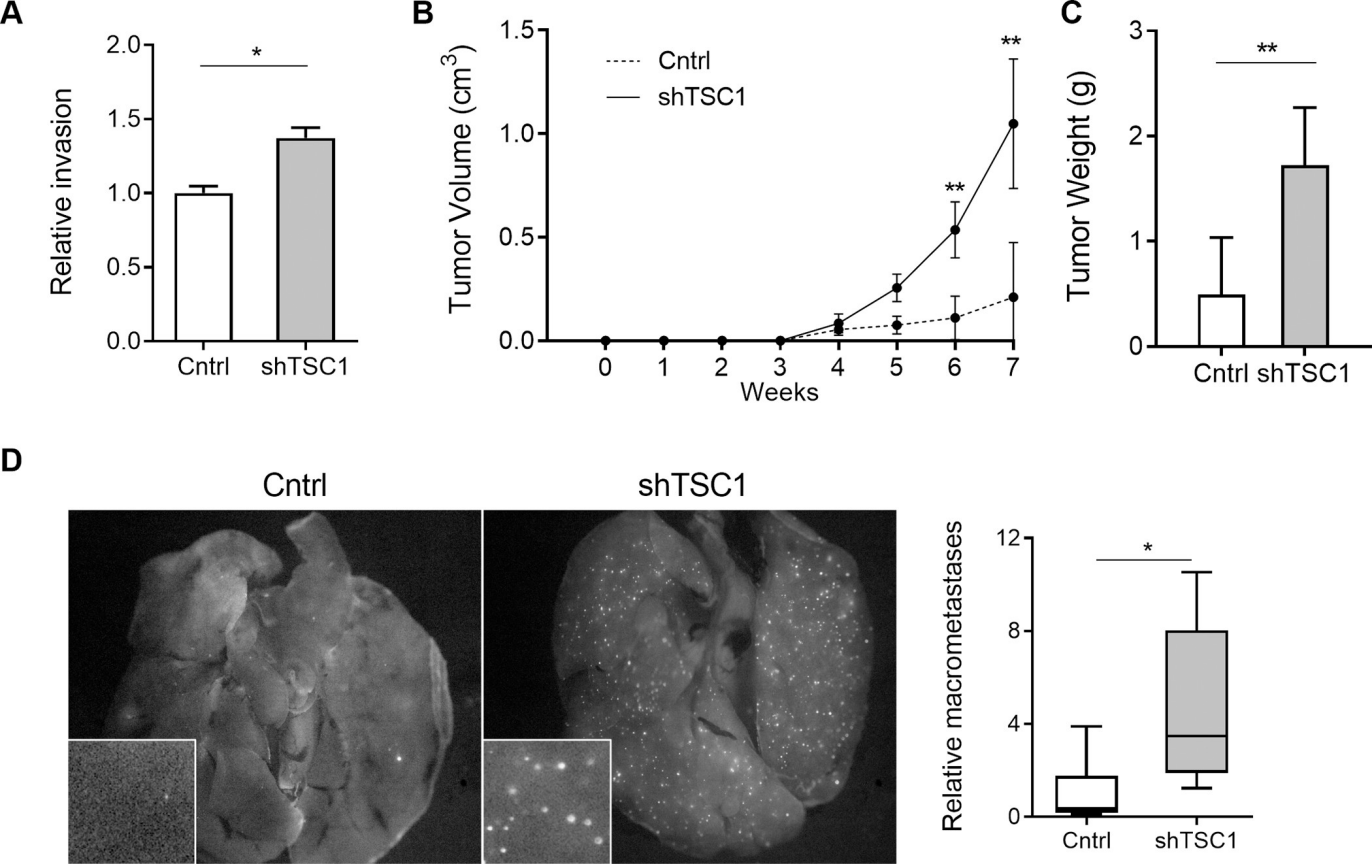
Independent silencing of *TSC1* promotes invasion and metastasis. (A) Relative invasion of LMS cells transduced with shTSC1 or non-target shRNA control (Cntrl) in transwell assay (n = 3). Cells were counted on membrane inserts after crystal violet staining. Cell counts were normalized to the control group. (B) Primary tumor growth following subcutaneous injection of control (n = 10) and sh*TSC1* (n = 10) transduced LMS1 cells in immunocompromised mice. (C) Mean weight of primary tumors at endpoint (7 weeks) for control (n = 6) and sh*TSC1* (n = 8) groups. (D) Fluorescent images of GFP positive nodules in whole lungs (*left panel*). Box plot (*right*) depicts average number of GFP+ lung macrometastases in 3 randomly chosen fields (*inset*) for Ctrl (n = 10) and sh*TSC1* (n = 10) xenografted tumors. Statistical analyses were performed by two-tailed students t-test; *P < 0.05, ** *P* < 0.005.

Since *TSC1* was directly repressed by miR-130b, shRNA-mediated *TSC1* knockdown was performed in LMS1 cells to determine if the pro-tumorigenic effects of miR-130b were replicated. Cells were stably transduced with dual expression lentiviral vectors encoding either a short hairpin RNA against *TSC1* (sh*TSC1*) or a non-target control, and GFP. Transduction of sh*TSC1* resulted in a ~50% reduction of *TSC1* mRNA levels compared to the non-target control vector (S3A, S3B Fig). *TSC1* knockdown had no effects on cell proliferation or colony formation (S3C, S3D Fig), but led to significantly enhanced invasion in transwell assays (*P* = 0.011) (Fig 4A), consistent with the effects of miR-130b overexpression (Fig 2A, S1B, S1E Fig). In addition, *TSC1* knockdown enhanced LMS cell migration in scratch assays (*P* < 0.05; sh*TSC1* relative to control) (S3E Fig).

To investigate whether *TSC1* knockdown also enhances LMS tumor growth *in vivo*, sh*TSC1*- and control vector-transduced LMS1 cells were xenotransplanted into immunocompromised mice. Similar to miR-130b overexpressing cells, sh*TSC1* cells exhibited greater tumor volume (*P* = 0.002) and tumor weight at endpoint (*P* = 0.001), compared to controls (Fig 4B and 4C, respectively). Moreover, *ex vivo* analysis of resected lungs from the same mice revealed a 4.7-fold increase in the number of GFP positive tumor foci (*P* = 0.02) (Fig 4D) supporting an increase in metastatic capacity following *TSC1* knockdown. These findings indicate that *TSC1* repression and miR-130b overexpression result in similar tumorigenic phenotypes in LMS cells, including increased invasion as well as enhanced *in vivo* tumor growth and metastasis. MiR-130b-enhanced LMS tumor growth and metastasis may therefore enhance tumor growth and metastasis in part via *TSC1*-dependent mechanisms.

### MiR-130b expression levels regulate LMS cell morphology and migratory behavior

**Fig 5:**
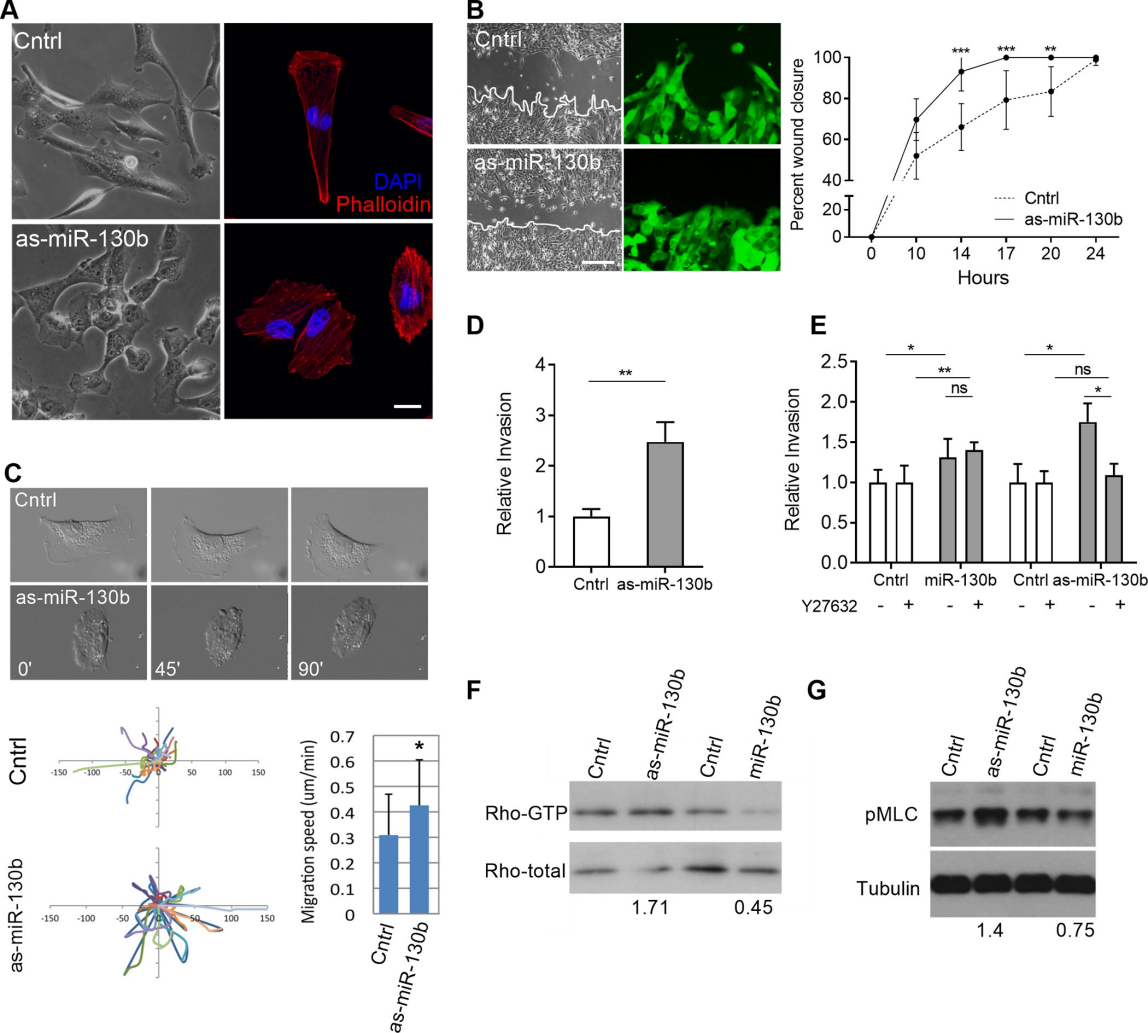
MiR-130b levels regulate LMS cell morphology, migratory and invasive behavior and Rho signaling. (A) Representative bright field (*left panels*) and immunofluorescence (*right panels*, red = phalloidin, blue = DAPI) images of LMS cells transduced with the anti-sense sequence of miR-130b (as-miR-130b) or control vector (Cntrl). Scale bar: 20 μm. (B) Scratch assay of control and as-miR-130b transduced LMS1 cells showing percentage of wound closure (relative to t = 0) over 24 h (n = 3). Representative bright field and fluorescent images (*left panels*) of scratch area showing prevalent leading-edge protrusions in control but not as-miR-130b transduced cells (arrowheads). Scale bar: 100 μm (C) Still images of time-lapse video microscopy of control and as-miR-130b transduced cells (*upper panels*), depicting a loss of lamellipodia and elongated cell morphology following miR-130b inhibition. Track plots indicating migration paths of individual cells (n = 25) from a single origin (center of axes) in control and as-miR-130b groups (*bottom panel*, *left*). Average migration speed (*bottom panel*, *right*) calculated by Image J. (D) Transwell assay showing relative invasion of as-miR-130b and control transduced LMS1 cells after 18 h. Cells were counted on membrane inserts after crystal violet staining. Cell counts were normalized to the control group. (E) Transwell assays showing relative invasion in the absence (-) or presence (+) of the ROCK inhibitor Y27632 (ROCKi). All invasion assays were performed in triplicate. Cell counts were normalized to the control groups without Y27632. (F) Rho-GTP levels in LMS1 cells by pull down assay following miR-130b down-regulation (as-miR-130b) and overexpression (miR-130b) relative to controls. Numbers indicate relative Rho-GTP band intensities of as-miR-130b and miR-130b groups versus control groups after normalization to total Rho levels. (G) Immunoblot of pMLC and tubulin in corresponding cell cultures. Numbers indicate relative pMLC band intensities of as-miR-130b and miR-130b groups versus control groups after normalization to tubulin levels. Statistical analyses were performed by two-tailed students t-test; *P < 0.05, ** *P* < 0.005, *** *P* < 0.001.

The pro-invasive and metastatic phenotype of miR-130b-overexpressing LMS1 cells suggests a capacity to regulate cell motility. To investigate this further, endogenous miR-130b levels in LMS1 cells were inhibited by transduction of a dual expression lentiviral vector encoding a short hairpin antisense sequence of miR-130b (as-miR-130b) and GFP (S4 Fig). MiR-130b repression did not affect LMS proliferation or colony forming potential (S4B, S4C Fig), but caused a prominent change in LMS1 cell morphology compared to control cells expressing a non-target shRNA (Fig 5A, S4A Fig). While control cells were elongated, with distinct directional lamellipodia-forefoot and lagging end, as-miR-130b transduced cells lost obvious orientation and appeared rounded (Fig 5A), with an increase in f-actin fibers, identified by phalloidin staining (Fig 5A, *right panels*). This morphological change was accompanied by an altered rate and mode of migration. In scratch assays, as-miR-130b cells closed the wound gap more rapidly (*P* < 0.001) (Fig 5B, *right panel*), and did not exhibit leading edge protrusions compared to controls (Fig 5B, *left panel*). Time-lapse microscopy further confirmed increased migration rates and non-lamellipodia driven movement (*P* < 0.05) (Fig 5C, S1, S2 Movies).

Compared to controls, as-miR-130b transduced LMS1 cells also exhibited enhanced invasive capacity in transwell assays (*P* = 0.0038) (Fig 5D). Since miR-130b overexpression was found to regulate the expression of Rho pathway effectors (Fig 3A), and Rho/ROCK signaling has been shown to mediate a rounded mode of cell motility [31], invasion assays were also performed in the presence of the Rho-associated protein kinase (ROCK) inhibitor Y27632, which prevents Rho activation. Y27632 treatment abolished the increased invasive capacity observed in as-miR-130b transduced cells, but not in miR-130b overexpressing cells (*P* = 0.046, Fig 5E). Furthermore, in as-miR-130b transduced cells, immunoblot revealed increased protein levels of the Rho active form and phosphorylated myosin light chain (pMLC)—a downstream readout of Rho activity (Fig 5F, 5G). Conversely, miR-130b overexpression decreased the levels of both proteins relative to controls, consistent with a Rho-independent mechanism of invasion (Fig 5F, 5G). Taken together, these observations indicate that miR-130b modulates LMS1 cell morphology and motility. Furthermore, cells can adopt differing modes of cell invasion depending on relative levels of the miRNA; endogenous miR-130b inhibition (as-miR-130b) induces a rounded, amoeboid mode of cell motility via Rho/ROCK dependent mechanisms, while elevated miR-130b levels promote cell movement independently of Rho signaling, consistent with an elongated morphology [31].

### MiR-130b overexpression and *TSC1* inhibition impair MSC differentiation along the SM lineage

**Fig 6:**
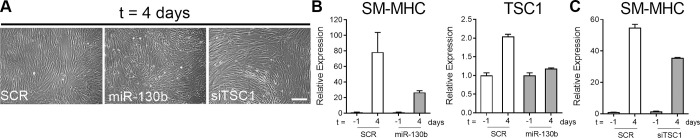
Smooth muscle cell (SMC) differentiation is impaired by miR-130b overexpression and *TSC1* repression. (A) Phase contrast images showing TXA_2_-mediated myogenic differentiation (t = 4 d) of primary hMSCs transfected with mimic control (SCR), miR-130b and siRNA targeting *TSC1* (siTSC1). (B) Relative mRNA levels of the SMC marker *SMMHC*, and *TSC1* at the 4 d endpoint following miR-130b transfection. (C) Relative mRNA levels of *SMMHC* at the 4 d endpoint following si*TSC1* transfection. Values are normalized to control SCR levels prior to myogenic induction (t = -1 d). Data are representative of 3 independent experiments. Scale bar: 100 μm.

In addition to its upregulation in LMS, miR-130b was also previously found to be downregulated during SM differentiation of MSCs *in vitro* [14]. To investigate whether miR-130b promotes an undifferentiated phenotype in SM, bone marrow-derived MSCs were transfected with miR-130b or SCR mimic controls and induced to differentiate into smooth muscle cells (SMC) with thromboxane A2 (TXA2), as previously described [14]. Following myogenic induction, increased miR-130b levels prevented the formation of neatly aligned parallel bundles of cells, commonly seen in fully differentiated SMC cultures, compared to miRNA mimic controls (Fig 6A). Defective cellular organization was accompanied by a reduction in the upregulation of *SMMHC* mRNA levels, indicating impaired SMC maturation (Fig 6B). Additionally, miR-130b transfection also impaired upregulation of *TSC1* mRNA levels during SM differentiation suggesting that *TSC1* is also a direct miR-130b target in this context (Fig 6B). Independent silencing of *TSC1* by transfection of small interfering RNAs (si*TSC1*) in hMSCs also resulted in impaired SMC maturation, indicated by a partial reduction of *SMMHC* mRNA levels in differentiating cultures compared to non-target siRNA controls (SCR) (Fig 6C). These data indicate that miR-130b can function as a negative regulator of SMC differentiation which may be mediated, in part, by direct repression of *TSC1*. Interestingly, we have previously shown that the shRNA-mediated silencing of *DICER1* also abrogates SMC differentiation [14]. Accordingly, we found that *DICER1* is a miR-130b target in LMS cells (S5 Fig). Thus, in addition to *TSC1* suppression, direct targeting of *DICER1* may also contribute to inhibition of SMC maturation by miR-130b. Together these findings provide further evidence linking miR-130b and *TSC1* repression to a poorly differentiated phenotype and enhanced tumorigenic capacity of LMS cells.

## Discussion

**Fig 7:**
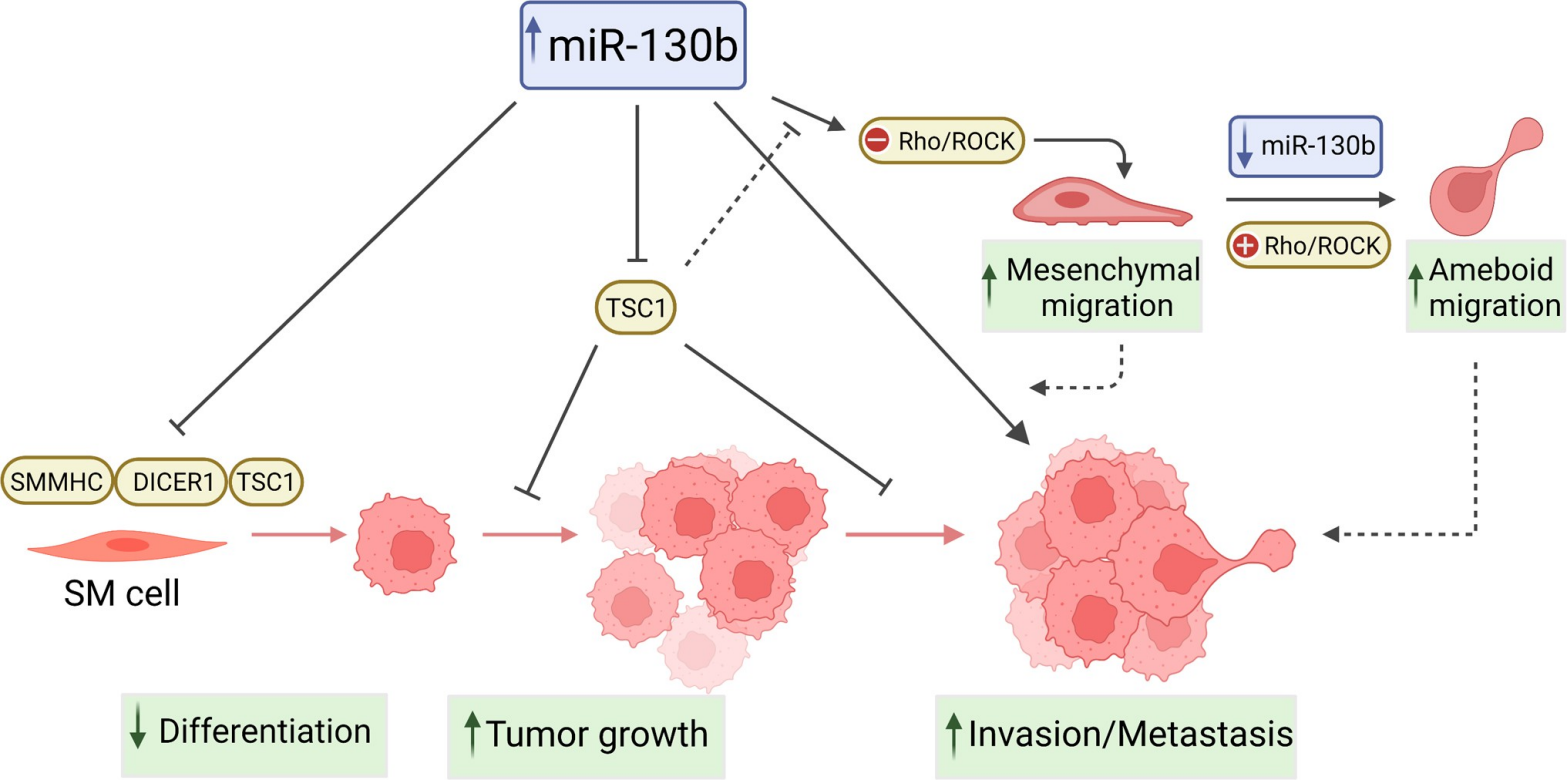
Proposed tumorigenic mechanisms of miR-130b in LMS. Solid lines indicate demonstrated interactions; dashed lines represent hypothetical associations with tumorigenic mechanisms.

This study shows miR-130b as a pro-oncogenic miRNA in LMS, which enhances *in vivo* tumor growth, cell invasion and metastatic potential, and inhibits SM differentiation. These findings are consistent with miRNA expression profiles that indicate miR-130b upregulation of in LMS relative to normal SM, and miR-130b downregulation during SM differentiation of MSCs [14]. MiR-130b effects may be in part due to repression of *TSC1*, which was also found to be differentially expressed in both LMS and SM differentiation, and a target of miR-130b. These observations indicate that miR-130b targets effectors of both, cellular transformation and differentiation, and may critically contribute to the undifferentiated and aggressive nature of LMS (Fig 7).

MiR-130b has been found to exert both tumor promoting and suppressive functions depending on the cancer type. Elevated miR-130b levels are associated with poor clinical prognosis in bladder cancer [32], and reduced patient survival in prostate cancer [33], hepatocellular carcinoma [34], colorectal carcinoma [35], Ewing sarcoma [36], osteosarcoma [37], non-small cell lung cancer [38], and lung adenocarcinoma [39]. Modulation of cellular miR-130b levels has led to regulation of several pro-oncogenic pathways, including NF-κb activation in bladder cancer [40], angiogenesis and EMT in colorectal carcinoma [35], as well as enhanced proliferation and invasion of glioma cells [41, 42] and hepatocellular carcinoma [43]. Additionally, miR-130b has been associated with drug resistance in renal cell carcinoma [44], ovarian [45] and breast cancer [46]. MiR-130b also inhibits tumor suppressor function in esophageal squamous cell carcinoma by repression of PTEN [47] and SASH1 [48], and in bladder cancer by targeting VGLL4 [49]. In hepatocellular carcinoma cells, miR-130b has been found to promote a proliferative and metastatic phenotype by targeting PTEN [50], or p53-induced nuclear protein-1 TP53INP1 [51].

Our data support a role for miR-130b in promoting the aggressiveness of LMS, in part by repressing *TSC1*, a tumor suppressor that controls anabolic cell growth and differentiation through inhibition of mammalian target of rapamycin (mTOR) [52]. Luciferase reporter assays revealed that miR-130b inhibits *TSC1* expression in LMS cells by directly binding to conserved sites at +4453 (S3) and +4879 (S4) of its 3’-UTR. Moreover, *TSC1* was significantly downregulated in LMS patient samples, and shRNA-mediated *TSC1* knockdown replicated the effects of miR-130b in cell invasion, *in vivo* tumor growth and metastasis. These observations further support TSC1 as a LMS tumor suppressor, and are consistent with AKT-mTOR pathway activation as a major oncogenic driver of LMS [53]. Accordingly, constitutive mTOR activation was found to be critical for LMS initiation and progression in conditional *SM22a-Pten* knockout mice [54].

MiRNA knockdown studies revealed that miR-130b also regulates cell morphology and motility of LMS cells. Stable miR-130b inhibition led to an amoeboid mode of movement characterized by loss of cell polarity, rounded morphology, increased migration and invasion that was abrogated by the Rho/ROCK inhibitor, Y27632. While miR-130b-overexpressing cells also displayed increased invasive capacity, consistent with their enhanced metastatic capacity *in vivo*, this process was Rho/ROCK-independent; characteristic of a mesenchymal mode of movement that uses elongated protrusions [31, 55]. Analysis of Rho-GTP and pMLC levels also revealed an inverse correlation with miR-130b, providing further evidence that miR-130b functions as a negative regulator of Rho signaling. Collectively, these data suggest that LMS cells exhibit plasticity in migration mechanisms in response to miR-130b levels, with elevated expression promoting mesenchymal movement, and low expression inducing rounded ameoboidal migration, via de-repression of Rho-associated targets. Luciferase reporter-3’UTR assays identified *TSC1*, the endocytosis regulator *RAB5A*, and the ADP-ribosylation factor guanine exchange factor, *ARFGEF1* (also known as BIG1), as miR-130b targets which can act as regulators of Rho activation and signaling [56, 57]. Indeed, the Rho-type GTPase Activating Protein 1, *ARHGAP1*, which was not found to be regulated by miR-130b in LMS cells (Fig 3B), has been identified as a direct target of miR-130b in Ewing Sarcoma, where upregulation of this miRNA leads to enhanced invasion and metastasis via stimulation of the CDC42/PAK1/AP-1 axis [58]. Further elucidation of miR-130b regulation of the tightly controlled Rho/Rac GTPase network is clearly warranted to identify relationships between cytoskeletal structure, migration, invasive capacity and modes of cell movement in LMS as well as other metastatic cancers.

In accordance with the undifferentiated, pleiomorphic appearance of miR-130b overexpressing LMS tumors, SM differentiation of MSCs was also impaired following overexpression of miR-130b. This may be in part due to repression of *TSC1* or *DICER1*, as independent silencing of both mRNAs was found to partially recapitulate miR-130b inhibitory effects (S5 Fig, and [14]). We have previously shown that conditional inactivation of *TSC1* in MSCs leads to impaired SM differentiation and a hyperproliferative phenotype accompanied by increased senescence and oxidative stress [59]. Similarly, amplification of the stem cell pool and blockade of cellular differentiation have been reported following *TSC1* loss via mTORC1 activation [60, 61]. *TSC1* repression by miR-130b in SM progenitors may lead to sustained proliferative signaling and acquisition or enhancement of an oncogenic phenotype, while also maintaining cells in an immature ‘undifferentiated’ state. Interestingly, thromboxane A2-induced SM myogenesis of MSCs is also mediated via Rho/ROCK signaling [28], providing further evidence of a shared miR-130b-regulatory network in LMS tumor progression and SMC differentiation.

In conclusion, our findings indicate that miR-130b is elevated in LMS, where it may accelerate tumor growth and metastasis through inhibition of the tumor suppressor, TSC1, and promotion of cell migration and invasion. Our data also provide evidence linking miR-130b to a poorly differentiated phenotype in SM and LMS tissues, via TSC1 repression, suggesting a common pathway in the processes of cellular differentiation and transformation. MiR-130b and the miR-130b/TSC1 axis may therefore provide novel molecular targets for therapeutic intervention to impede tumor growth and metastatic progression of poorly differentiated LMS.

## Supporting information

S1 Methods (DOCX)

S1 Table qPCR primers used for mRNA quantification and determination of gene amplification of the miR-130b locus. (XLSX)

S2 Table DNA sequences of truncated TSC1 3’UTR constructs for luciferase reporter assays. DNA sequences of truncated *TSC1* 3’UTR constructs for luciferase reporter assays. Putative miR-130b binding sites are indicated in bold. Mutated sequence is shown in red. Sequence locations are indicated relative to the beginning of the 3’UTR.
(XLSX)

S1 Fig Effects of miR-130b overexpression on LMS1 cell growth, *in vitro*. (A) Relative miR-130b expression levels, measured by RT-qPCR, in LMS cells (LMS1) stably transduced with a lentiviral construct containing miR-130b sequence (miR-130b) relative to control cells transduced with a non-targeting miRNA (Cntrl). (B) Growth curves of miR-130b- or control-transduced LMS1 in monolayer cultures in media with serum (S) (solid lines) and under serum-free conditions (SF) (hashed lines). Proliferation was assessed by crystal violet staining and measuring absorbance at 595 nm. (C) Cell survival under attachment-free conditions. Transduced cells were plated on ultra-low attachment plates, forming cell clusters which were dissociated and counted every 24 h for 3 days. Representative bright field images for each group are shown under graph. (D) Scratch assay of control and miR-130b transduced LMS1 showing percentage of wound closure (relative to t = 0) over 24 h. Representative bright field images showing extent of wound closures over time are shown under graph (*lower panels*). (E) Colony forming capacity of control and miR-130b-transduced LMS1 cells, 9 days after plating. Images (*lower panels*) show crystal violet-stained colonies at endpoint. (F) Representative images of miR-130b- and control-transduced cells cultured as sarcospheres in methyl cellulose. At day 7, cells were isolated from a subset of each group and single cell suspensions were reseeded in methyl cellulose to assess for self-renewal. Data represent mean ± SD of triplicate measurements; experiments were performed in 2 stably transduced LMS1 cell lines. Scale bars: 100 μM.
(TIF)

S2 Fig Luciferase activities of S3 and S4 *TSC1*-3’UTR constructs following inhibition of endogenous miR-130b levels in UT-1 cells. Values represent luciferase units in UT-1 cells of wild type or mutant S3 and S4 constructs following mock (no inhibitor), control (ctrl HI) or miR-130b (miR-130b HI) hairpin inhibitor cotransfection. *P < 0.05, vs Ctrl HI. Values are normalized to the mock controls for each luciferase construct. Data represent mean ± SD, n = 4. Note that miR-130b inhibition did not significantly increase luciferase activity of the wild type S4 construct or either of the mutant S3 and S4 constructs.
(TIF)

S3 Fig Effects of independent silencing of TSC1 on LMS *in vitro* cell proliferation and migration. (A) Representative bright field (*top panels*) and fluorescence images (*bottom panels*) indicating transduction efficiency of LMS1 cells transduced with dual expression lentiviral vectors encoding shTSC1or non-target shRNA (Cntrl), and GFP. (B) Relative *TSC1* expression levels in LMS1 cells in control and knockdown groups, measured by RT-qPCR. (C) Growth curves of shTSC1 and control-transduced LMS1 cells cultured in standard growth media (10% serum). (D) Colony forming capacity of control and shTSC1-transduced LMS1 cells, 9 days after plating. Images (*lower panels*) show crystal violet-stained colonies at endpoint. (E) Scratch assay of control and shTSC1 transduced LMS1 showing percentage of wound closure (relative to t = 0) over 24 h. Representative bright field images showing extent of wound closures over time are shown. Data represent mean ± SD of triplicate measurements; experiments were performed in 2 stably transduced LMS1 cell lines. Scale bars: 100 μM. Statistical analyses were performed by two-tailed students t-test; * *P* < 0.05, ** *P* < 0.005.
(TIF)

S4 Fig LMS1 cell proliferation and migration following inhibition of endogenous *miR-130b*. (A) Representative bright field (*top panels*) and fluorescence images (*bottom panels*) indicating transduction efficiency of LMS1 cells transduced with dual expression lentiviral vectors encoding the antisense sequence of miR-130b (as-miR-130b) or non-target RNA control sequence (Cntrl), and GFP. Scale bar: 100 μM. (B) Colony forming capacity of control and as-miR-130b-transduced LMS1 cells, 9 days after plating. Images (*lower panels*) show crystal violet-stained colonies at endpoint. (C) Growth curves of as-miR-130b and control cells grown in standard growth media (10% serum). Data represent mean ± SD of triplicate measurements; experiments were performed in 2 stably transduced LMS1 cell lines.
(TIF)

S5 Fig DICER1 is a direct target of miR-130b. (A) Relative expression levels of *DICER1*, measured by RT-qPCR, in UT1 and LMS1 cells transfected with miR-130b or control (SCR) oligonucleotide mimics. (B) Western blot analysis of DICER1 protein expression in miR-130b and control mimic transfected cells. (C) Luciferase-3’UTR reporter assay for validation of DICER1 as a direct target of miR-130b. Values represent relative luciferase units (RLU) in 293T cells transfected with a luciferase expression vector containing the 3’UTR of *DICER1* without mimics (-), scrambled control (SCR), a non-targeting mimic (NT), or miR-130b mimic. Values are normalized to the mock (-) control group (= 1). Data represent mean ± SD, n = 3–5. * *P* < 0.05 vs all control groups.
(TIF)

S1 Movie Time-lapse video microscopy of an LMS1 cell transduced with a lentiviral vector expressing a non-target control shRNA. A representative control cell is shown exhibiting an elongated morphology and directional lamellopodia driven movement.
(MOV)

S2 Movie Time-lapse video microscopy of an LMS1 cell transduced with a lentiviral vector expressing a short hairpin antisense sequence of miR-130b (as-miR-130b). A representative as-miR-130b modified cell is shown, exhibiting a rounded ameboid morphology and non-lamellopodia driven movement.
(MOV)

S1 Raw images (PDF)
