# Supplementary material for: MiR-130b modulates the invasive, migratory, and metastatic behavior of leiomyosarcoma
Source: PLoS One. 2023 Jan 26;18(1):e0278844. doi: 10.1371/journal.pone.0278844 (PMC9879492; doi:10.1371/journal.pone.0278844)
Supplement: S1 Methods — (DOCX) [file pone.0278844.s001.docx]

**Supplementary Methods**

**Proliferation assay**

Cell were plated in 96-well plates (3 x 10^3^ cells/well) and cultured in IMDM with or without 10% FBS for up to 10 days. Cells fixed with 0.1% gluteraldehyde at the indicated timepoints and stained with 1% crystal violet. For quantification, the stain was recovered from the cultures by was dissolving with 15% acetic acid and absorbance measured at 595 nm using a plate reader.

**Colony-forming and sarcosphere assays**

*Colony-forming assay:* Colony-forming assays were performed by seeding cells into 6-well plates (0.5 - 2 × 10^3^ cells) in IMDM with 10% FBS. Medium was replaced every 3 days, and after 9 days cells were fixed and stained with 1% crystal violet. After extensive washing, colonies were counted. Triplicate wells were counted for each group. *Sarcosphere Assay:* To identify cells capable of forming stem-cell like spheres, cells were seeded into suspension cultures using a modification of previous methods (1, 2). Cells were plated at varying densities in 12-well low-cell adhesion plates in serum-free DMEM/F12 medium containing B-27 supplement, 1% BSA, human EGF-1 (10 ng/ml) and human bFGF (10 ng/ml) mixed with an equal volume of 2% methylcellulose (Sigma). Fresh EGF-1 and bFGF aliquots were added every other day. At the indicated time points, colonies (>50 cells) were imaged by inverted phase contrast microscopy and quantitated.

**References**

1. Gibbs CP, Kukekov VG, Reith JD, Tchigrinova O, Suslov ON, Scott EW, et al. Stem-like cells in bone sarcomas: implications for tumorigenesis. Neoplasia. 2005;7(11):967-76. PubMed PMID: 16331882. PMCID: PMC1502023.

2. Fujii H, Honoki K, Tsujiuchi T, Kido A, Yoshitani K, Takakura Y. Sphere-forming stem-like cell populations with drug resistance in human sarcoma cell lines. Int J Oncol. 2009;34(5):1381-6. PubMed PMID: 19360350.
