# Supplementary figures and images for: MiR-130b modulates the invasive, migratory, and metastatic behavior of leiomyosarcoma
Source: PLoS One. 2023 Jan 26;18(1):e0278844. doi: 10.1371/journal.pone.0278844 (PMC9879492; doi:10.1371/journal.pone.0278844)

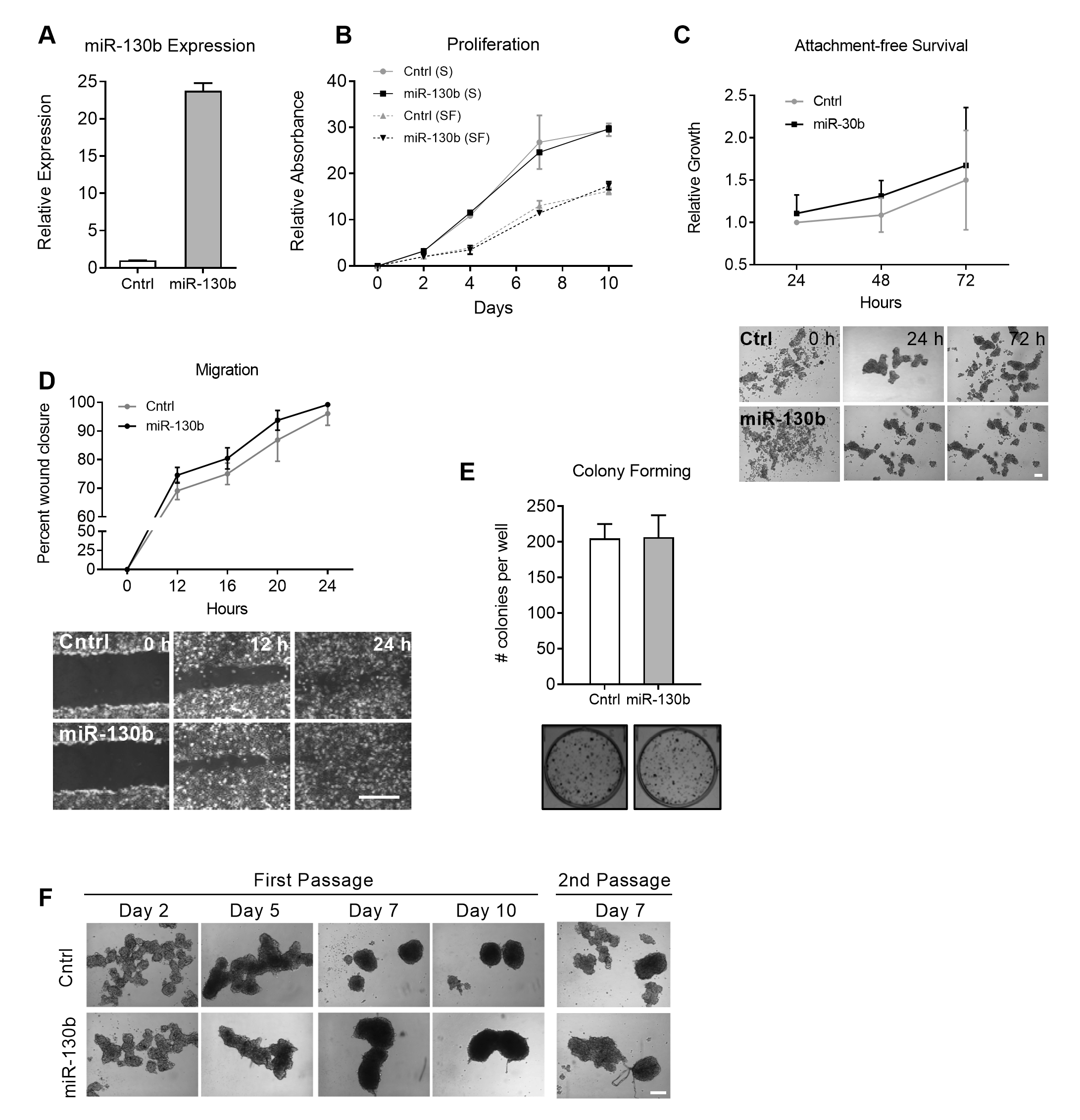

Supplement: S1 Fig — (A) Relative miR-130b expression levels, measured by RT-qPCR, in LMS cells (LMS1) stably transduced with a lentiviral construct containing miR-130b sequence (miR-130b) relative to control cells transduced with a non-targeting miRNA (Cntrl). (B) Growth curves of miR-130b- or control-transduced LMS1 in monolayer cultures in media with serum (S) (solid lines) and under serum-free conditions (SF) (hashed lines). Proliferation was assessed by crystal violet staining and measuring absorbance at 595 nm. (C) Cell survival under attachment-free conditions. Transduced cells were plated on ultra-low attachment plates, forming cell clusters which were dissociated and counted every 24 h for 3 days. Representative bright field images for each group are shown under graph. (D) Scratch assay of control and miR-130b transduced LMS1 showing percentage of wound closure (relative to t = 0) over 24 h. Representative bright field images showing extent of wound closures over time are shown under graph (lower panels). (E) Colony forming capacity of control and miR-130b-transduced LMS1 cells, 9 days after plating. Images (lower panels) show crystal violet-stained colonies at endpoint. (F) Representative images of miR-130b- and control-transduced cells cultured as sarcospheres in methyl cellulose. At day 7, cells were isolated from a subset of each group and single cell suspensions were reseeded in methyl cellulose to assess for self-renewal. Data represent mean ± SD of triplicate measurements; experiments were performed in 2 stably transduced LMS1 cell lines. Scale bars: 100 μM. (TIF) [file pone.0278844.s004.tif]

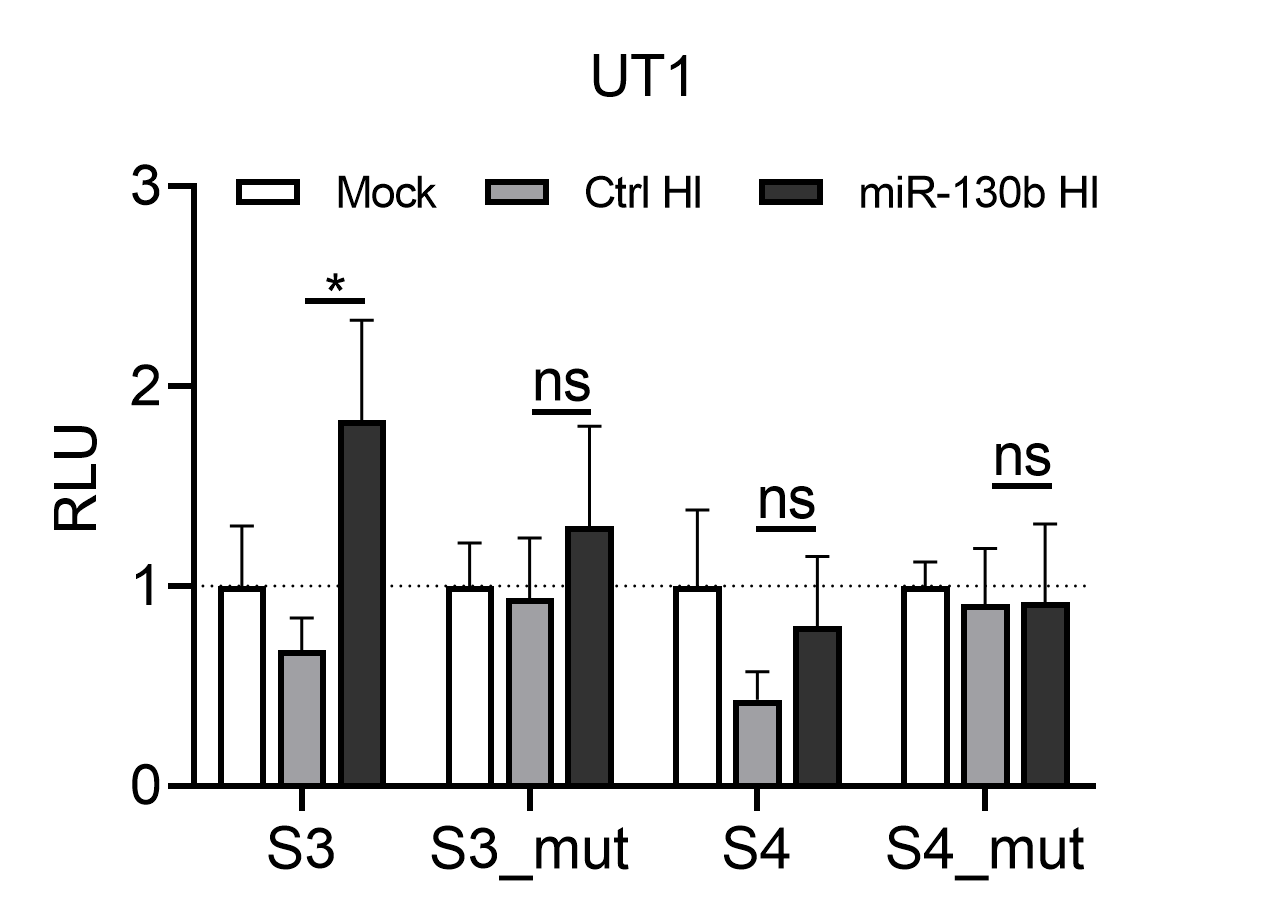

Supplement: S2 Fig — Values represent luciferase units in UT-1 cells of wild type or mutant S3 and S4 constructs following mock (no inhibitor), control (ctrl HI) or miR-130b (miR-130b HI) hairpin inhibitor cotransfection. *P < 0.05, vs Ctrl HI. Values are normalized to the mock controls for each luciferase construct. Data represent mean ± SD, n = 4. Note that miR-130b inhibition did not significantly increase luciferase activity of the wild type S4 construct or either of the mutant S3 and S4 constructs. (TIF) [file pone.0278844.s005.tif]

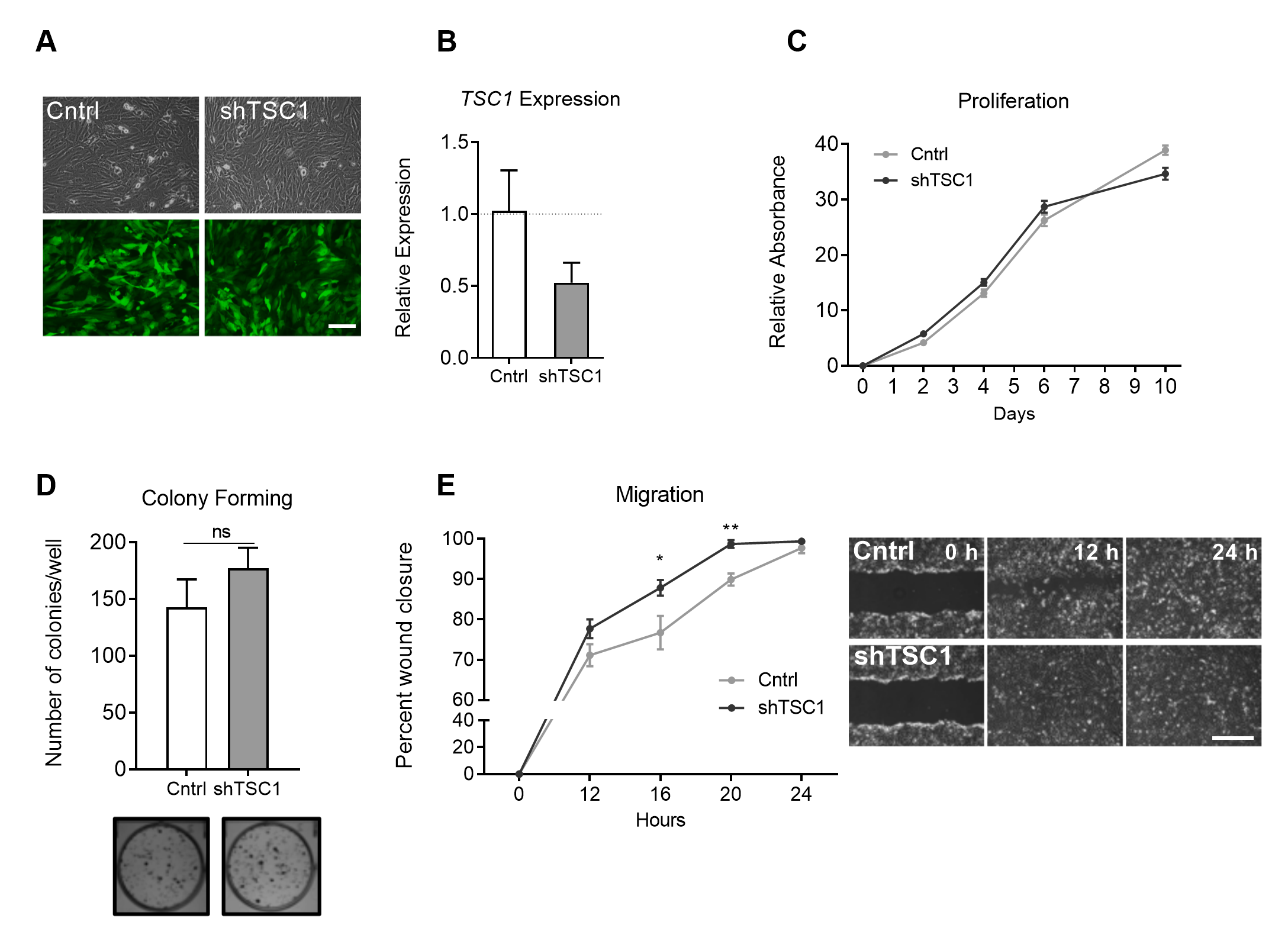

Supplement: S3 Fig — (A) Representative bright field (top panels) and fluorescence images (bottom panels) indicating transduction efficiency of LMS1 cells transduced with dual expression lentiviral vectors encoding shTSC1or non-target shRNA (Cntrl), and GFP. (B) Relative TSC1 expression levels in LMS1 cells in control and knockdown groups, measured by RT-qPCR. (C) Growth curves of shTSC1 and control-transduced LMS1 cells cultured in standard growth media (10% serum). (D) Colony forming capacity of control and shTSC1-transduced LMS1 cells, 9 days after plating. Images (lower panels) show crystal violet-stained colonies at endpoint. (E) Scratch assay of control and shTSC1 transduced LMS1 showing percentage of wound closure (relative to t = 0) over 24 h. Representative bright field images showing extent of wound closures over time are shown. Data represent mean ± SD of triplicate measurements; experiments were performed in 2 stably transduced LMS1 cell lines. Scale bars: 100 μM. Statistical analyses were performed by two-tailed students t-test; * P < 0.05, ** P < 0.005. (TIF) [file pone.0278844.s006.tif]

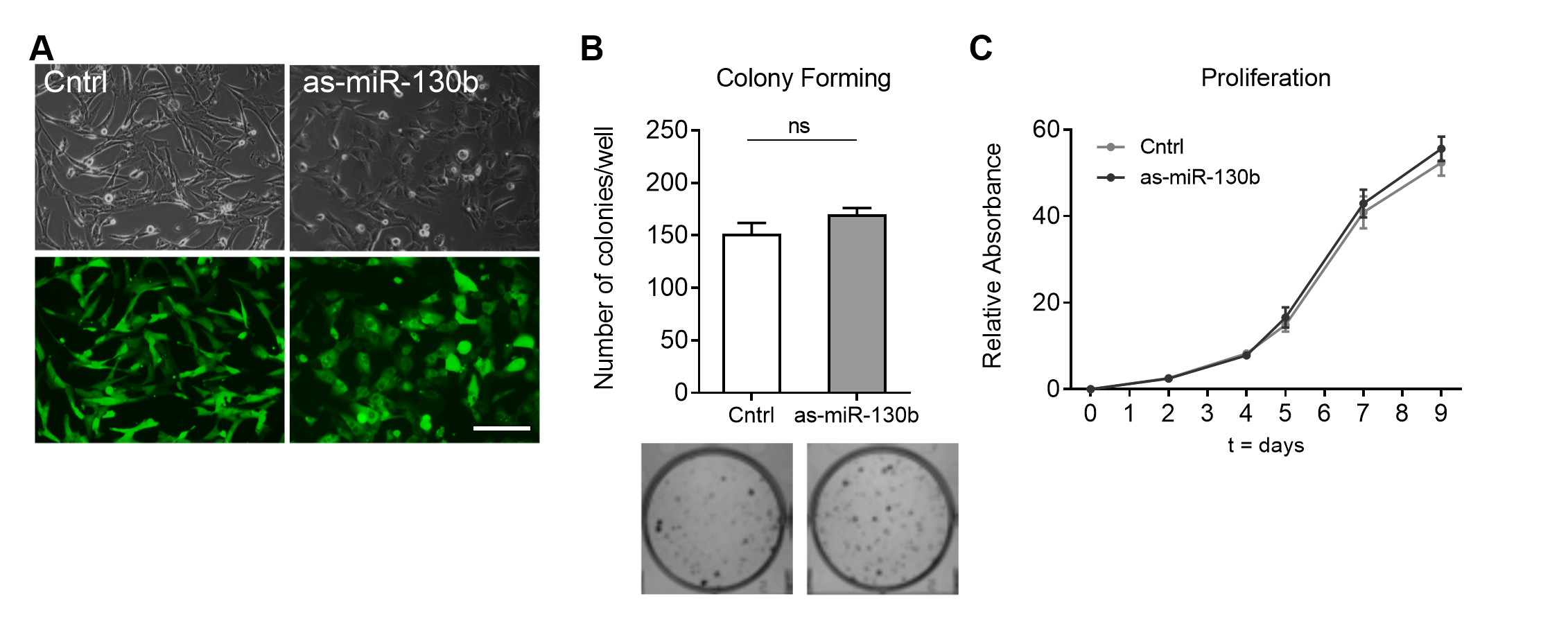

Supplement: S4 Fig — (A) Representative bright field (top panels) and fluorescence images (bottom panels) indicating transduction efficiency of LMS1 cells transduced with dual expression lentiviral vectors encoding the antisense sequence of miR-130b (as-miR-130b) or non-target RNA control sequence (Cntrl), and GFP. Scale bar: 100 μM. (B) Colony forming capacity of control and as-miR-130b-transduced LMS1 cells, 9 days after plating. Images (lower panels) show crystal violet-stained colonies at endpoint. (C) Growth curves of as-miR-130b and control cells grown in standard growth media (10% serum). Data represent mean ± SD of triplicate measurements; experiments were performed in 2 stably transduced LMS1 cell lines. (TIF) [file pone.0278844.s007.tif]

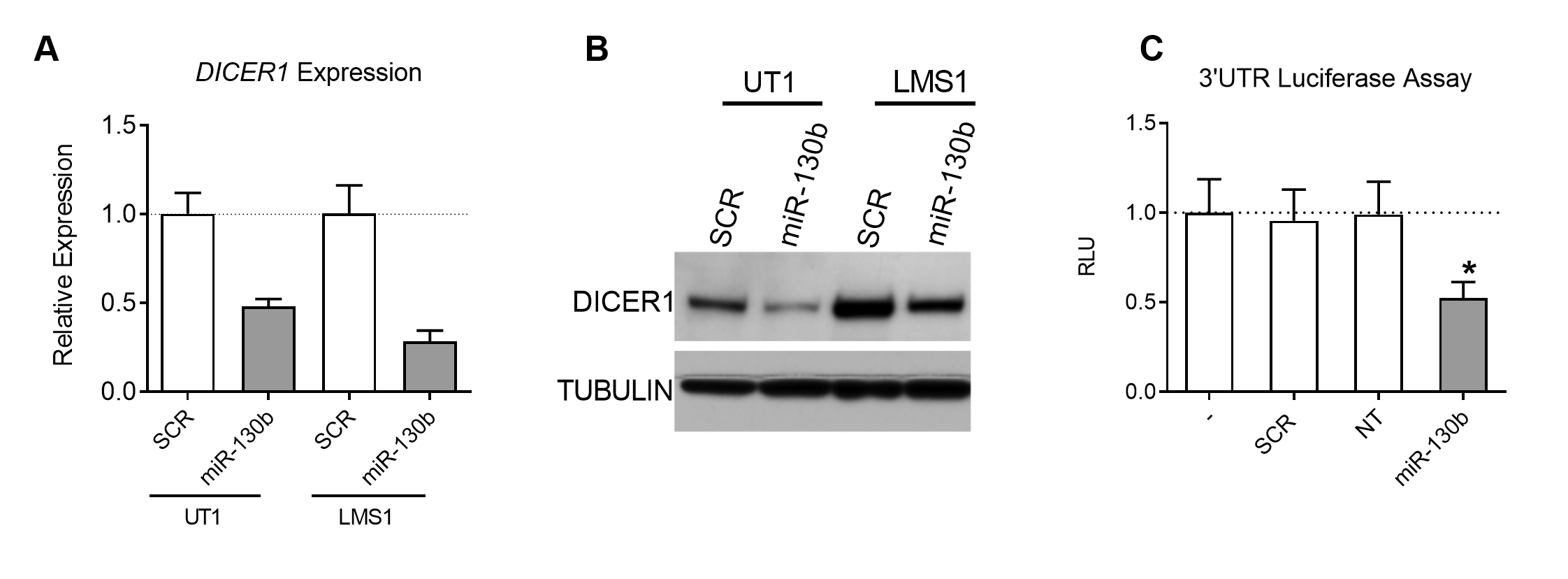

Supplement: S5 Fig — (A) Relative expression levels of DICER1, measured by RT-qPCR, in UT1 and LMS1 cells transfected with miR-130b or control (SCR) oligonucleotide mimics. (B) Western blot analysis of DICER1 protein expression in miR-130b and control mimic transfected cells. (C) Luciferase-3’UTR reporter assay for validation of DICER1 as a direct target of miR-130b. Values represent relative luciferase units (RLU) in 293T cells transfected with a luciferase expression vector containing the 3’UTR of DICER1 without mimics (-), scrambled control (SCR), a non-targeting mimic (NT), or miR-130b mimic. Values are normalized to the mock (-) control group (= 1). Data represent mean ± SD, n = 3–5. * P < 0.05 vs all control groups. (TIF) [file pone.0278844.s008.tif]
