# Supplementary material for: MiR-130b modulates the invasive, migratory, and metastatic behavior of leiomyosarcoma
Source: PLoS One. 2023 Jan 26;18(1):e0278844. doi: 10.1371/journal.pone.0278844 (PMC9879492; doi:10.1371/journal.pone.0278844)

S1 Raw Images

Unadjusted blot images for Fig. 3D. Load order (L-R; boxed region): 1. SCR 2. miR-130b 3. SCR 4. miR-130b. Images are film exposures ranging from 20 s to 2 min.

TSC1:

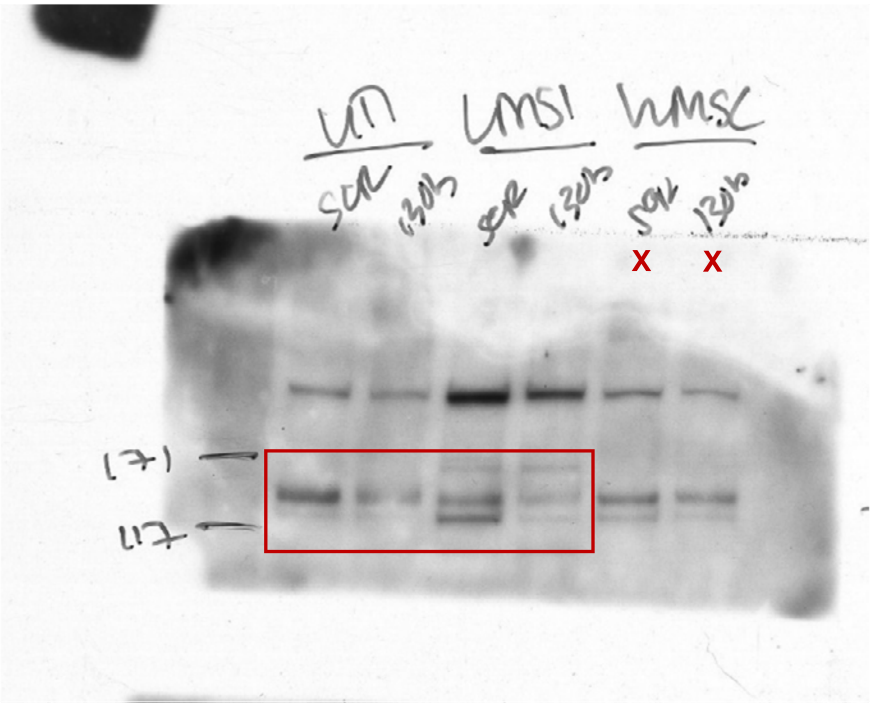

Tubulin:

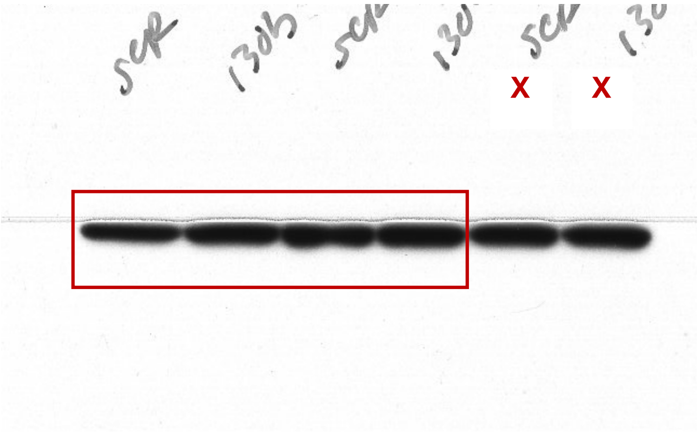

Unadjusted blot images for Fig. 5D. Load order (L-R; boxed region): 1. Cntrl 2. as-miR-130b 3. Cntrl 4. miR-130b. Images are film exposures ranging from 20 s to 2 min.

Rho GTP:

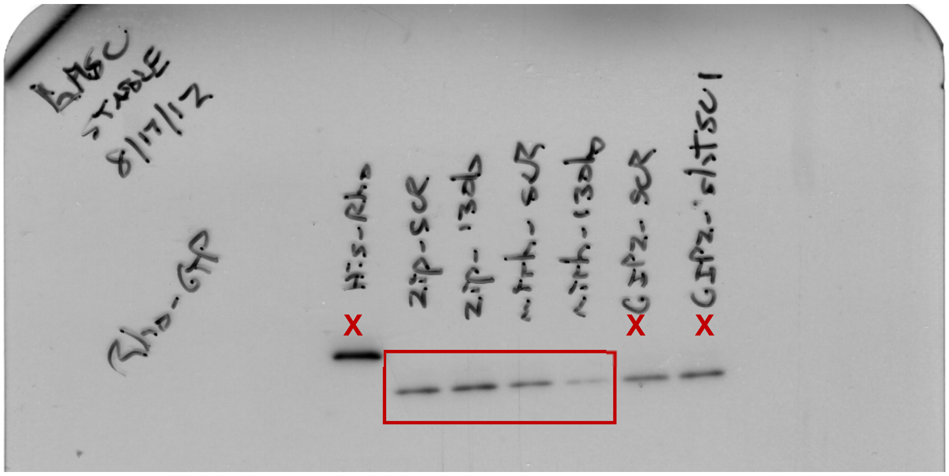

Rho-total:

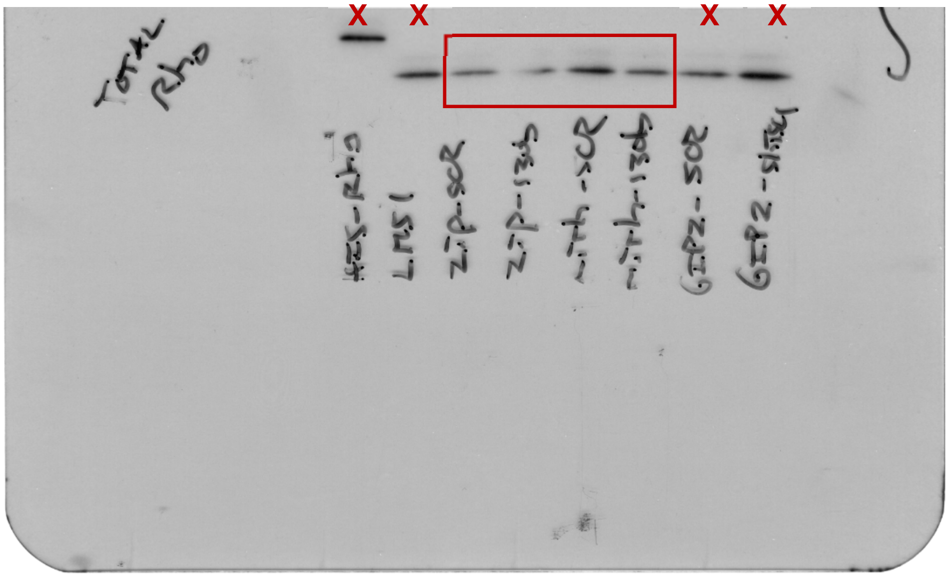

Unadjusted blot images for <u>Supp Fig. 5B.</u> Load order (L-R; boxed region): 1. SCR 2. miR-130b 3. SCR 4. miR-130b. Images are film exposures ranging from 20 s to 2 min.

DICER1:

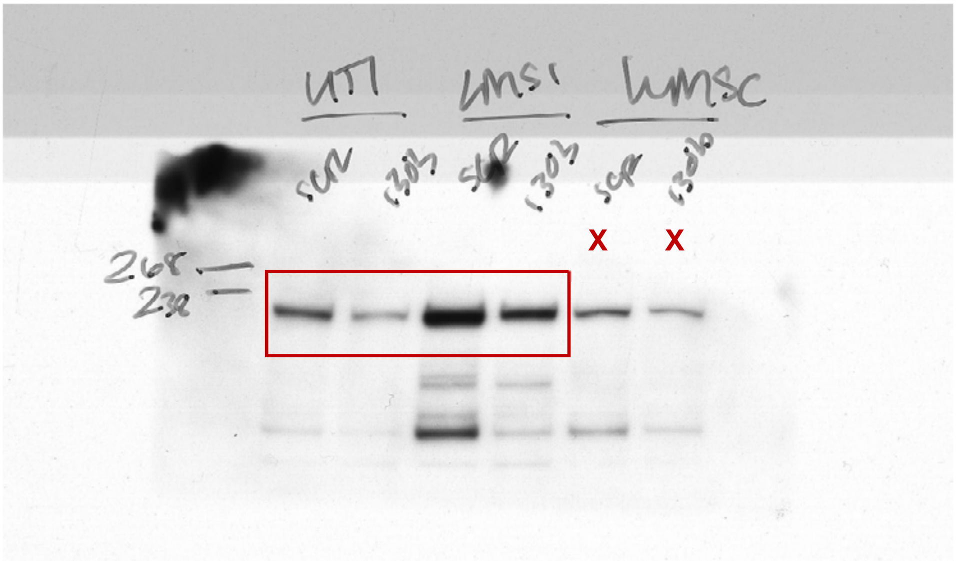

Tubulin:

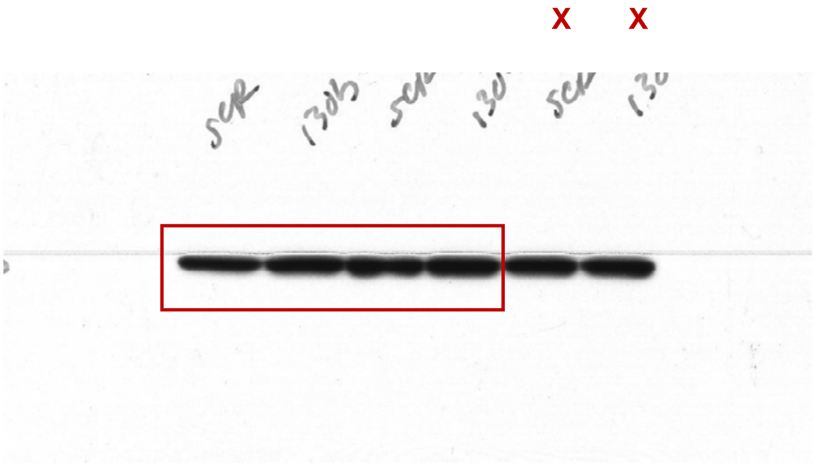

Supplement: S1 Raw images — (PDF) [file pone.0278844.s011.pdf]
